# Tribochemical nanolithography: selective mechanochemical removal of photocleavable nitrophenyl protecting groups with 23 nm resolution at speeds of up to 1 mm s^−1^†

**DOI:** 10.1039/d2sc06305k

**Published:** 2023-01-16

**Authors:** Robert E. Ducker, Oscar Siles Brügge, Anthony J. H. M. Meijer, Graham J. Leggett

**Affiliations:** a Department of Chemistry, University of Sheffield Brook Hill Sheffield S3 7HF UK Graham.Leggett@sheffield.ac.uk

## Abstract

We describe the mechanochemical regulation of a reaction that would otherwise be considered to be photochemical, *via* a simple process that yields nm spatial resolution. An atomic force microscope (AFM) probe is used to remove photocleavable nitrophenyl protecting groups from alkylsilane films at loads too small for mechanical wear, thus enabling nanoscale differentiation of chemical reactivity. Feature sizes of 20–50 nm are achieved repeatably and controllably at writing rates up to 1 mm s^−1^. Line widths vary monotonically with the load up to 2000 nN. To demonstrate the capacity for sophisticated surface functionalisation provided by this strategy, we show that functionalization of nanolines with nitrilo triacetic acid enables site-specific immobilization of histidine-tagged green fluorescent protein. Density functional theory (DFT) calculations reveal that the key energetic barrier in the photo-deprotection reaction of the nitrophenyl protecting group is excitation of a π–π* transition (3.1 eV) *via* an intramolecular charge-transfer mechanism. Under modest loading, compression of the adsorbate layer causes a decrease in the N–N separation, with the effect that this energy barrier can be reduced to as little as 1.2 eV. Thus, deprotection becomes possible *via* either absorption of visible photons or phononic excitation transfer, facilitating fast nanolithography with a very small feature size.

## Abstract 2

Photolithography without optics: compression of nitrophenyl protecting groups under an atomic force microscope probe modifies their electronic structure and reduces the energy barrier to deprotection, enabling nanolithography without UV light.

## Graphical abstract

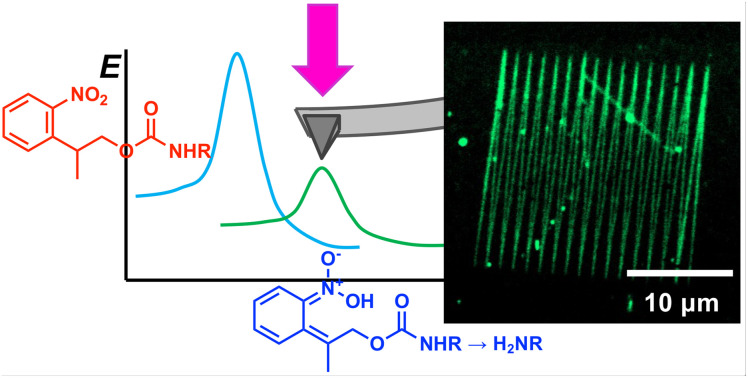

## Introduction

In molecular nanoscience, the integration of top-down (lithographic) and bottom-up (synthetic chemical) fabrication methods is a central challenge. One approach is to utilize photochemical methods; the synthetic chemistry literature is replete with photochemical methods for the control of chemical reactivity,^1–4^ and as long ago as 1991, Fodor *et al.* described an approach to the integration of top-down and bottom-up methods at the micrometre scale, which they called “light-directed chemical synthesis”.^5,6^ They prepared aminosilane films with nitroveratryloxycarbonyl (NVOC) protecting groups; on exposure to near-UV light, the film was quantitatively deprotected, allowing derivatization with an amino acid that was itself protected by an NVOC group.^5^ By carrying out a sequence of such deprotection and reaction steps, they were able to fabricate arrays of peptides, in which a different peptide was synthesised at each location in each array.

Near-field methods enable the translation of photochemical methods to the nanometre scale.^7–9^ In self-assembled monolayers (SAMs), near-field lithographic techniques have been found to yield line widths as small as 9 nm.^10^ A wide variety of approaches has been described including the patterning of monolayers on metal and oxide supports^11^ and the direct modification of films of nanoparticles, fullerenes and polymers.^9^ Parallel fabrication has been demonstrated.^12^ A closely related approach, beam-pen lithography, has been developed by Mirkin and co-workers.^13–16^ In this approach, an array of pyramidal probes is formed on a transparent polymer slab and coated with an opaque metal. Apertures are formed at the apices of these probes to enable them to act as near field light sources.

However, near-field methods rely on instrumentation that is not readily available, and the resolution of beam-pen lithography is typically of the order of hundreds of nm. Thus, neither offers a simple, generic method for the control of photochemical reactions with a spatial resolution better than 100 nm. Here, we describe a different approach to the integration of top-down and bottom-up methods in which a photocleavable protecting group is removed *via* a mechanochemical mechanism, using a widely available commercial atomic force microscope (AFM), to yield small linewidths with high repeatability at speeds up to 1 mm s^−1^.

In recent years there has been an explosion of interest in mechanochemistry,^17–19^ the mechanical activation of chemical reactivity.^19–31^ In bulk phases, mechanical grinding enables solventless mechanochemical synthesis,^21,32^ attracting growing interest as a means to mitigate the environmental impact of chemical processes.^33^ At interfaces, tribochemical processes are known to play a role in determining the performance of lubrication systems. For example, zinc dialkyldithiophosphate (ZDDP) is added to engine lubricants to reduce wear and to improve performance.^34–36^ A wide variety of other kinds of interfacial films undergo tribochemical modification, including inorganic surfaces,^37–41^ carbonaceous materials^42–44^ and layers of molecular adsorbates,^45–47^ and mechanical loading can change the distribution of products in a chemical reaction. For example, Biswas *et al.* showed that under mechanical loading, the *syn* product predominates over the *anti* conformer in the photodimerisation of acenaphthylene, reversing the behaviour observed in the absence of mechanical loading.^48^ Atomic force microscopy has proved to be a powerful tool for the investigation of tribochemical modification,^49^ and has also been used to modify surfaces at the nanometre scale *via* utilization of the energy dissipated in the tip-sample contact.^19,50–52^

**Scheme 1:**
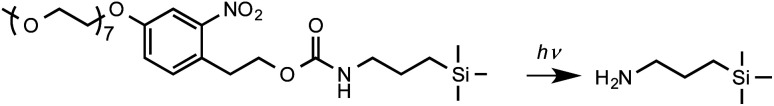
Photodeprotection of OEG-NPOC-APTES to yield an amine-functionalised film.

Here we examine the use of a mechanochemical process to drive a very specific chemical transformation: the selective removal of photocleavable protecting groups in nm regions of space. Inspired by the work of Fodor *et al.* on light-directed chemical synthesis,^5^ previous work in this laboratory has utilized aminosilanes with photocleavable nitrophenyl protecting groups for near-field lithography.^53^ These are prepared *via* convenient synthetic pathways, and offer robust protection with quantitative removal on exposure to UV light. For example, (nitrophenyl)propyloxycarbonyl (NPPOC) protected alkylsilane films were used in parallel near-field lithography experiments in which features with linewidths of 125 nm were written over an area 1.5 mm wide and utilised to form polymer nanostructures^12^ and (nitrophenyl) ethoxycarbonyl (NPEOC) protected aminosilanes were derivatized with hepta(ethylene glycol) derivatives to render them protein resistant enabling the production of protein nanostructures using near-field methods.^54,55^ Once the film has undergone deprotection (Scheme 1), proteins may be adsorbed directly to nanopatterned amine groups, enabling the creation of multiplexed protein nanopatterns when OEG-NPEOC-APTES is used in conjunction with near-field lithography.^56^

Here we show that the deprotection reaction shown in Scheme 1 can be driven using an AFM probe in the absence of a UV light source under a load too small for mechanical bond-breaking. Thus, the use of nitrophenyl groups as mechanochemical protecting groups is facilitated, enabling the regulation of chemical reactivity at nm spatial resolution. DFT calculations indicate that the energy barrier for photocleavage of nitrophenyl protecting groups is greatly reduced under modest loading, enabling selective deprotection with exquisite resolution. This mechanochemical protecting group strategy is compatible with subsequent synthetic elaboration by a broad range of methods,^5,6,12,53–59^ illustrated here by demonstrating the site-specific binding of histidine-tagged proteins. This lithographic strategy is capable of implementation on any commercial AFM system, and facile synthetic routes are available to silanes with nitrophenyl protecting groups, making this a methodology that is capable of widespread adoption.

## Results and discussion

### Lithographic process

**Fig. 1:**
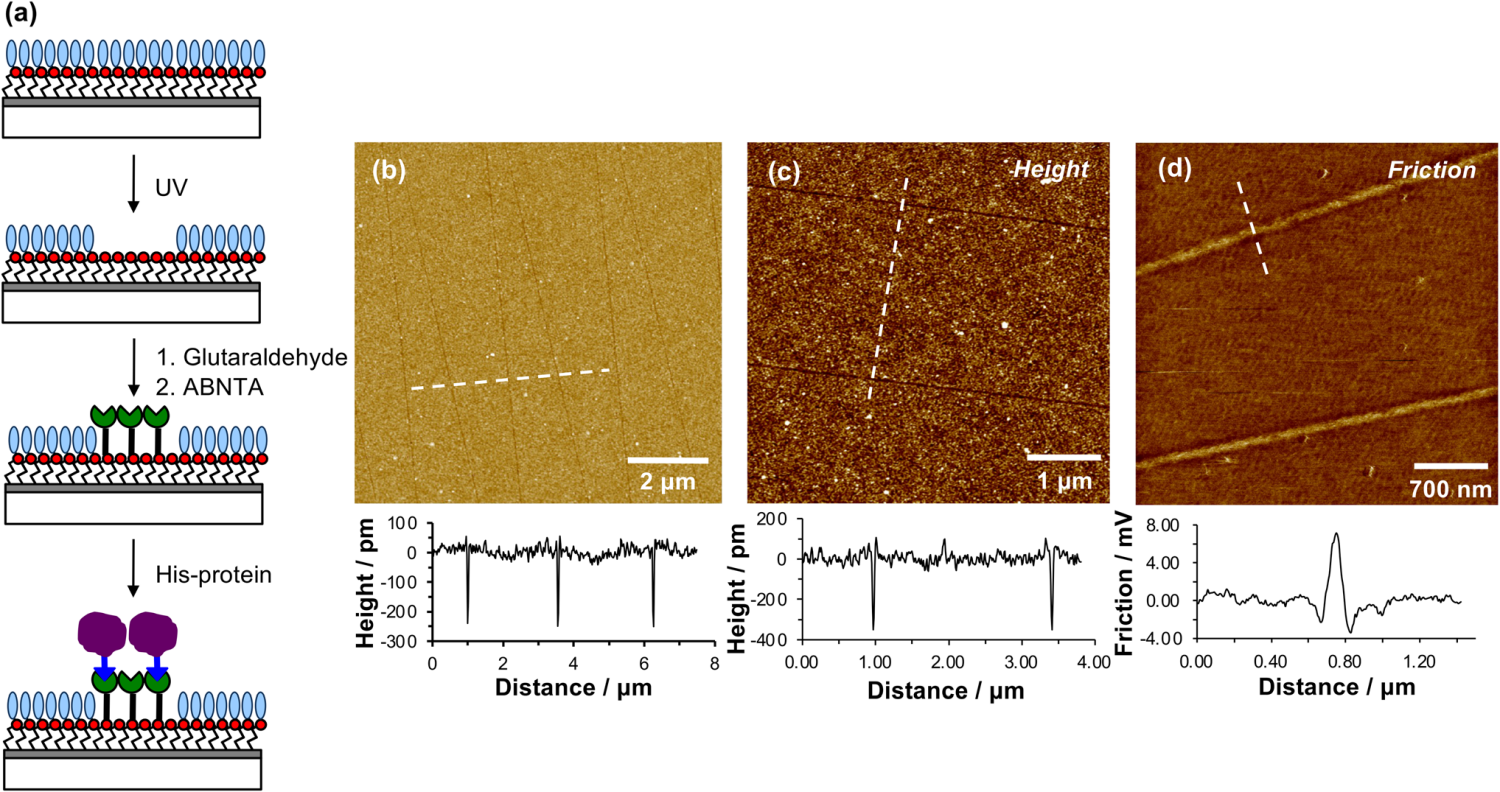
(a) Schematic diagram showing the photo-deprotection of OEG-NPEOC-APTES, and the derivatization of deprotected amine groups using glutaraldehyde, ABNTA/Ni^2+^ and His-tagged protein. (b and c) AFM tapping mode topographical images of patterns formed by tracing an AFM probe across an OEG-NPEOC-APTES film at a load of 500 nN and speed of 1 μm s^−1^. Line sections along the dashed lines are shown underneath the images. (d) Friction force microscopy image of nanolines, together with a line section along the dashed line.

When a tapping mode AFM probe (nominal radius of curvature 7–10 nm) is traced across NPPOC-APTES and OEG-NPEOC-APTES films at modest loads, narrow, well-defined lines are formed. Fig. 1b and c show tapping mode topographical images of features formed at a load of 500 nN in films formed by the adsorption of OEG-NPEOC-APTES onto glass. The lines in Fig. 1b have a full width at half maximum (FWHM) of 30 nm and depths of 300 to 350 pm. These data suggest that the lithographic process causes removal of material from the surface. We hypothesised that this is attributable to removal of the OEG-NPEOC protecting group.

To investigate the nature of the lithographic process, patterns were characterized by friction force microscopy (FFM). Nanolines display brighter contrast in FFM images than the surrounding surface (Fig. 1d), indicating a larger friction force in those areas than on unmodified regions of the sample. The surface free energies of OEG-NPEOC-APTES and APTES are similar, thus surface energy changes do not account for the differential friction contrast. Rather, the increase in friction after lithographic modification probably indicates that removal of the protecting groups is incomplete under the conditions used here, yielding a film of reduced density that offers more pathways for energy dissipation than the intact, close-packed film.

Nanolithography was carried out for loads up to 19 000 nN. For loads less than 200 nN, no removal of material was observed. At loads greater than 200 nN, the depth of the features was found to increase with the load, from 103 ± 8 pm at a load of 200 nN to 572 ± 16 pm at a load of 5000 nN. However, for loads in the range 5000–15 000 nN, the feature depth did not increase further. When loads greater than 15 000 nN were used, broad features were formed. However, on subsequently reducing the load, narrow features could no longer be fabricated, suggesting the tip had become irreversibly blunted at these very high loads.

To test whether nanopattern formation occurred *via* mechanical wear of the surface, behaviour was compared for APTES films at loads between 500 nN and 15 000 nN (ESI†). For these silanes that did not have a photoremovable protecting group, patterning was not observed at a load of 500 nN, but it was observed on increasing the load above 12 500 nN, suggesting that there was a very high load threshold for mechanical wear. Thus, the patterning process observed for OEG-NPEOC-APTES films at lower loads is not attributable to mechanical wear.

**Fig. 2:**
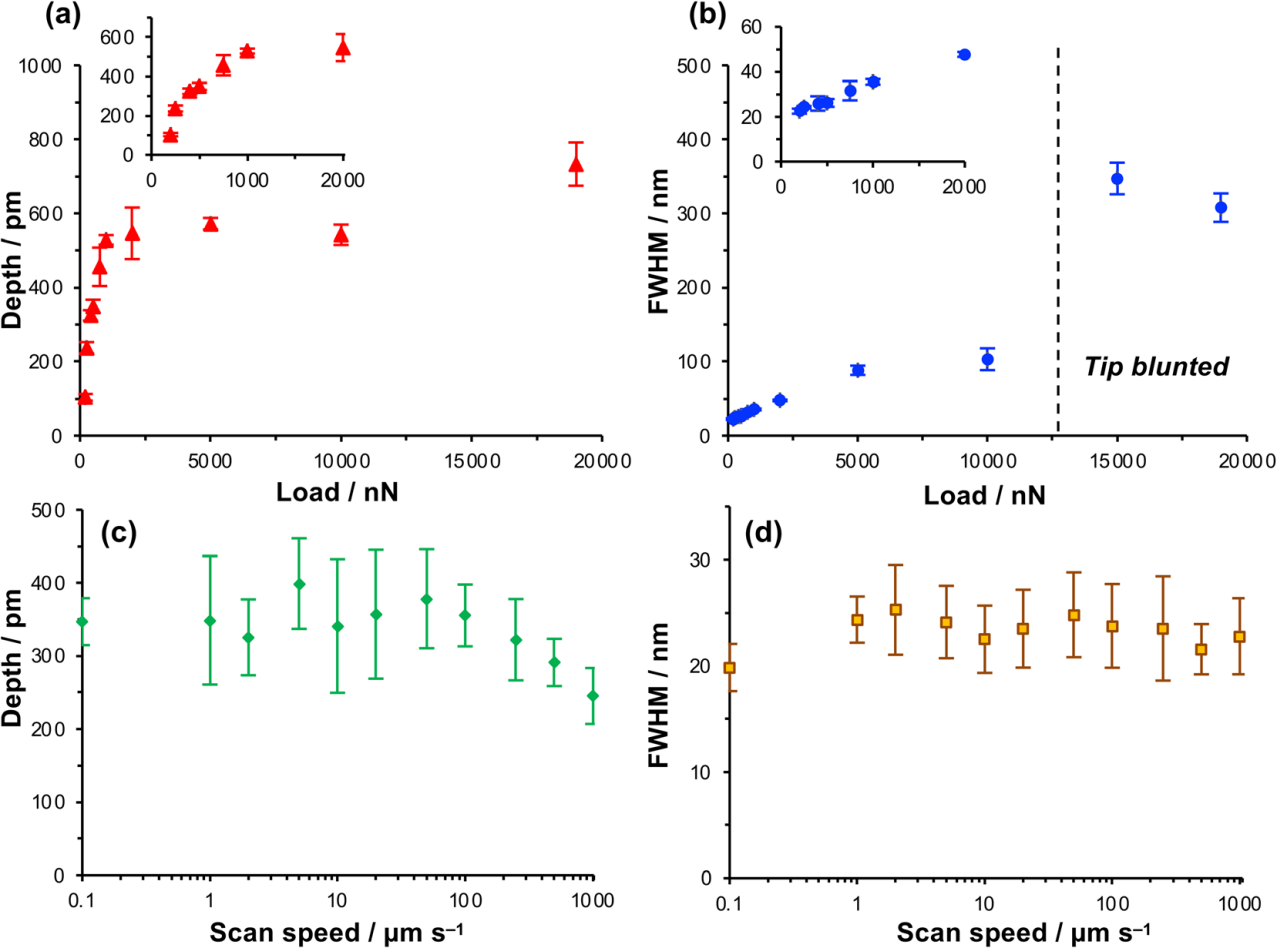
(a) Variation in the depth of nanolines with the normal load. (b) Variation in the full width at half maximum depth (FWHM) with the normal load. (c and d) Variation in, respectively, the depth and FWHM of nanolines as a function of the writing speed at a load of 1000 nN.

The depth of the nanolines produced lithographically increased rapidly as a function of load up to loads of ∼1000 nN, but thereafter the depth increased more slowly, approaching a limiting value at a load of ∼5000 nN (Fig. 2a). In contrast, the FWHM of the features increased monotonically as a function of the load up to a load of 5000 nN (Fig. 2b), but thereafter the rate of increase in FWHM with load declined. At 200 nN, the FWHM was 22.5 ± 1.1 nm, increasing to 26.2 ± 1.7 nm at a load of 500 nN and 31.6 ± 4.2 nm at 750 nN. These feature sizes are, for molecular layers, remarkably small and the small standard deviations indicate that the process is well-controlled and repeatable.

Increasing the load has two effects. First, the contact area increases as a function of the load. The increase in FWHM as a function of the load in Fig. 2b is consistent with a progressive increase in the tip-sample contact area. Second, the friction force increases as the load increases. Friction is a dissipative phenomenon, thus the energy available for bond-breaking in the tip-sample contact area is expected to increase as the load increases.

To test whether the depths of the nanolines might be correlated with the rate of energy dissipation in the tip-sample contact, the feature depth and the FWHM were measured as a function of the tip speed over a range from 0.1 μm s^−1^ (the lowest practicable writing rate on our instrument) to 1000 μm s^−1^ (Fig. 2c and d) at a constant load of 1000 nN. Friction forces are expected to increase with the sliding rate;^60–62^ thus if the lithographic process depends on the rate of energy dissipation in the contact area, the feature depth should also depend on the writing rate. However, the data in Fig. 2c and d show that neither the feature depth nor the line width vary significantly with the sliding speed over a large range. The widths of the features produced by the lithographic process are 24 ± 5 nm at tip speeds from 0.1 μm s^−1^ to 1.0 mm s^−1^. Up to a writing speed of 100 μm s^−1^, one to two orders of magnitude higher than typically used in scanning probe lithography of molecular films, there is no significant change in the feature depth as the writing rate is increased (Fig. 2c). Eventually, at very high rates (above 100 μm s^−1^), the feature depth is found to decrease slightly.

The absence of a correlation between the feature widths and the writing rate suggests that there is not a direct relationship between the lithographic process and energy dissipated in frictional interactions. At high speeds there is a reduction in the feature depth, although it should be noted that the errors are large for the feature depth at lower speeds. Moreover, this reduction in feature depth is suggestive of less effective dissipation at the tip-sample contact, rather than increased dissipation. Thus, these data further support the conclusion that the lithographic process here does not involve mechanical wear; an alternative explanation is required.

### Removal of the nitrophenyl group enables formation of protein nanostructures

We hypothesized that under loading, the silane film is compressed leading to an alteration of the energy barrier for removal of the nitrophenyl protecting group. Unmodified OEG-NPEOC-APTES surfaces are protein-resistant; thus, if the lithographic process causes deprotection of amines, it should be possible to derivatize them in a selective fashion. To test this experimentally, we treated patterned surfaces with glutaraldehyde, which binds to amines, and then with aminobutyl(nitrilo triacetic acid) (ABNTA). Introduction of NTA functional groups in this way would allow complexation with Ni^2+^ enabling site-specific binding of His-tagged green fluorescent protein (GFP). Thus, any regions from which the protecting group had been selectively removed would be characterized by green fluorescence.

**Fig. 3:**
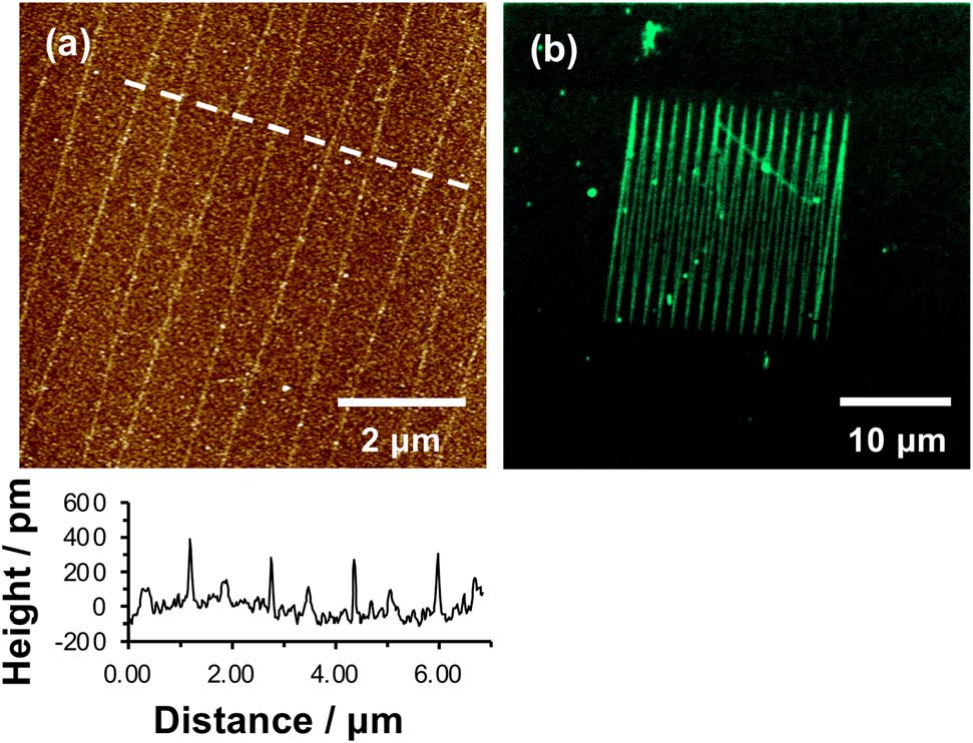
(a) AFM tapping mode topographical image of nanolines after treatment with glutaraldehyde, ABNTA and His-tagged GFP. (b) Confocal fluorescence microscopy image of the pattern shown in (a).

Fig. 3a shows an AFM topographical image of nanopatterns formed by tracing an AFM probe cross an OEG-NPEOC-APTES surface and derivatizing it as described. While the patterned structures appear as narrow trenches after formation (Fig. 1b and c), it is notable that after derivatization with GFP, they appear as features ∼350 pm high (Fig. 3a). The FWHM of the protein features in Fig. 3a was 50 nm.

Given that the height of GFP lying flat on a surface would be estimated to be ∼2.4 nm, and that the maximum feature depth formed lithographically was 572 pm, the observed height here is less than expected. There may be a number of explanations. First, GFP may undergo some conformational change on attachment to the surface. Second, and more likely, the feature depths measured after nanolithography (Fig. 2a) may be an underestimate because of the small feature widths: the features are of similar size to the probes (tip diameter ∼20 nm) with the consequence that there may be a convolution of the surface and tip contours for these small features, causing an over-estimate of the FWHM of the as-formed lines and an under-estimate of their depths.

Fluorescence microscopy performed on the same sample imaged in Fig. 3a yielded clear contrast between nanolines and other regions (Fig. 3b). The GFP-functionalized nanolines (bright green contrast) are clearly differentiated from the surrounding unmodified surface (dark contrast). The clarity of the image is the more remarkable given the very high aspect ratio of these lines, which have widths of 50 nm and lengths of 20 μm.

While these data demonstrate removal of the OEG group they do not unequivocally demonstrate site-specific binding of the protein. To demonstrate that binding occurred in a site-specific fashion, necessitating prior removal of the whole protecting group to enable derivatization of exposed amine groups, we used imidazole to reverse protein binding. Imidazole displaces His tags from the Ni^2+^ at the centre of the NTA-His tag complex.
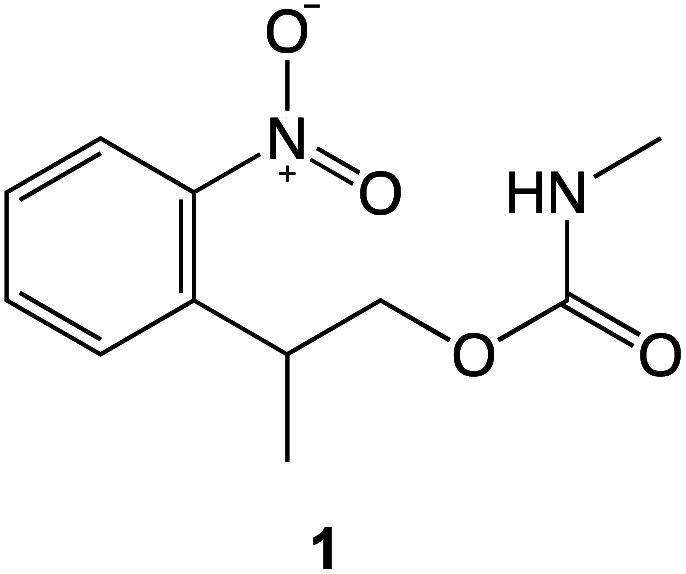


**Fig. 4:**
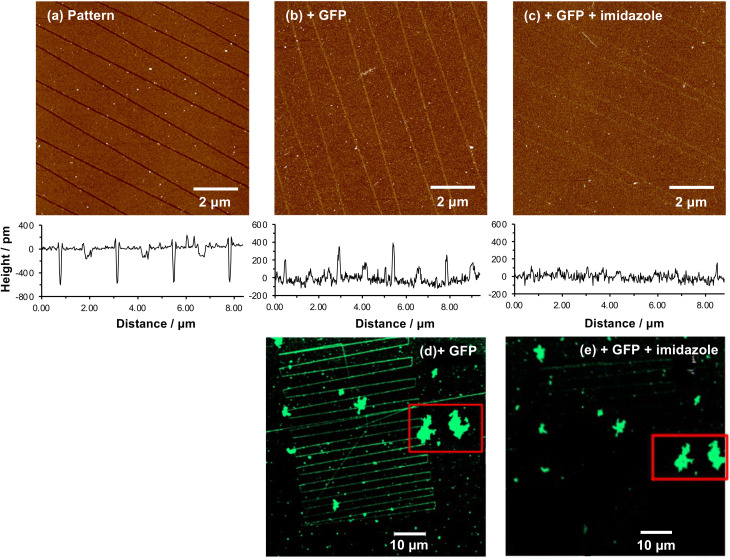
Site-specifically bound His-GFP is displaced by imidazole. (a–c) Atomic force microscopy images of a single sample (a) following nanolithography; (b) following reaction with glutaraldehyde and ABNTA, complexation with Ni^2+^ and binding of His-GFP; and (c) following treatment with imidazole. Representative line sections are shown under each image. (d and e) Confocal fluorescence microscopy images of His-GFP attached to nanopatterns before (d) and after (e) treatment with imidazole. Aggregates of protein, identified with a red box, are used to provide registry marks to enable relocation of the same surface region after treatment with imidazole.

Fig. 4a shows a series of nanolines formed as described above. The lines are trenches with depths of ∼500 pm. Fig. 4b shows the same lines after treatment with glutaraldehyde, ABNTA, Ni^2+^ and His-GFP. It can be seen that the nanolines are now higher than the surrounding, protein-resistant surface. The feature heights are ∼300 pm. However, after treatment with imidazole, the heights of these structures are found to be dramatically reduced (Fig. 4c). The nanolines may be observed faintly, although they are hard to identify in line sections suggesting their height is small. It may be that a low density of protein remains after treatment with imidazole (some non-specifically bound protein may be present). Alternatively, the derivatized amine groups (to which glutaraldehyde and ABNTA have been attached) may remain just visible as raised features.

A similar experiment was conducted using fluorescence microscopy. Fig. 4d shows a confocal fluorescence image of a large number of nanolines after derivatization and attachment of His-GFP. This image also exhibits a number of large features that are attributed to contamination (probably aggregates of protein). These features are useful because they provide registry marks at the surface. Two particular features are highlighted with a red box. The sample shown in Fig. 4d was treated with imidazole and returned to the microscope. The features marked with the red box were located. In Fig. 4d, nanolines derivatized with GFP are clearly visible. However, after treatment with imidazole (Fig. 4e) these lines have largely disappeared, confirming disruption of the NTA-His complex. Some lines are visible very faintly in the upper portion of Fig. 4e, probably attributable to a very low density of non-specifically bound protein.

### Compression of the nitrophenyl group causes a key energy barrier to be reduced, facilitating ambient deprotection

The preceding data provide clear evidence that under modest loading, selective removal of nitrophenyl protecting groups occurs to expose free amine groups. Thus, nanoscale control of chemical reactivity is facilitated with high resolution and high repeatability. The absence of a dependence of feature depth and width on sliding rates up to 100 μm s^−1^ (Fig. 2c and d) suggests that frictional energy dissipation is not rate-limiting; the process does not arise from mechanical wear causing cleavage of covalent bonds. Rather, these data suggest that compression of the sample causes a change in the activation barrier to removal of the nitrophenyl group, thus facilitating deprotection in the absence of a UV illumination source.

To test this hypothesis we carried out calculations using density functional theory (DFT), to investigate the effect of compression on the electronic structure of the nitrophenyl protecting group. Because there is a wealth of spectroscopic data on the deprotection of amines protected by NPPOC groups, we used a truncated form of NPPOC-APTES 1 in our calculations. This allowed us to validate our methodology by comparing calculated spectra with spectroscopic data. We reasoned that the additional methyl group close to the carbamate group in NPPOC is unlikely to play a significant role in deprotection.

Experimental data in our laboratory show that NPPOC-APTES and OEG-NPEOC-APTES have very similar photochemistry, consistent with similar deprotection mechanisms.^53–55^ The absence of lithographic modification of APTES films at modest loading also suggests that the propyl chain in OEG-NPEOC-APTES is not involved in the nanopatterning mechanism, so the calculations were further simplified by not modelling the effect of compression on the propyl chain.

**Fig. 5:**
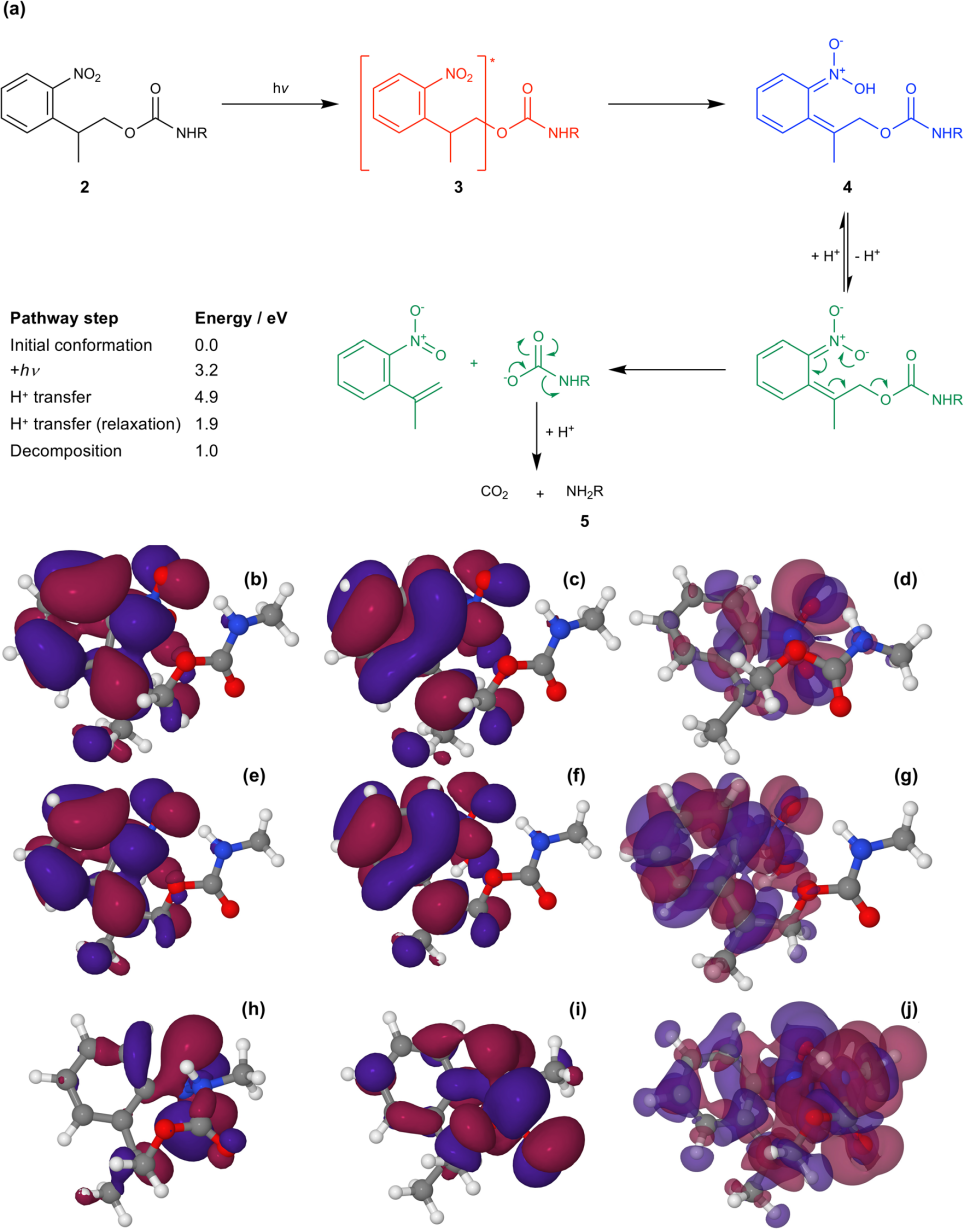
(a) Mechanism for the photo-deprotection of NPPOC-APTES. The table gives the energies of the key steps in the deprotection pathway (see ESI† for further detail). (b & c) HOMO and LUMO for NPPOC in the ground state. (d) Transition density associated with the S_1_ transition. (e & f) HOMO and LUMO for NPPOC after H-transfer in the optimized S_1_ state. (g) Transition density for the S_1_ transition for NPPOC after H-transfer. (h & i) HOMO and LUMO for the strained S_1_ state. (j) Transition density for the strained S_1_ transition.

Fig. 5a shows the mechanism of photo-deprotection based on the current consensus of the literature on this system.^58,63^ Absorption of a photon by the protected amine 2 causes a π–π* transition (S_0_ → S_1_) to yield the excited state 3. In the excited state, intramolecular proton transfer occurs to yield an acid-nitro intermediate 4. Loss of hydrogen from the acid-nitro species is followed by fragmentation of the NPPOC group leading to deprotection of the amine of APTES (5).

A number of density functionals and basis sets were compared, by testing their ability to reproduce the absorption spectrum on the NPPOC functional group (ESI†). The ω-B97XD functional^64^ was selected, together with the 6-311++G(d,p) basis set,^65,66^ which includes a diffuse function in order to better represent orbitals further from the nucleus, including those of lighter atoms. Two optimized ground-state configurations were determined from the DFT calculations (ESI†),^67,68^ described as “straight” and “folded”. The folded conformation had a lower energy (by 0.12 eV) than the “straight” conformation and was thus used as the starting conformation for subsequent calculations.

**Fig. 6:**
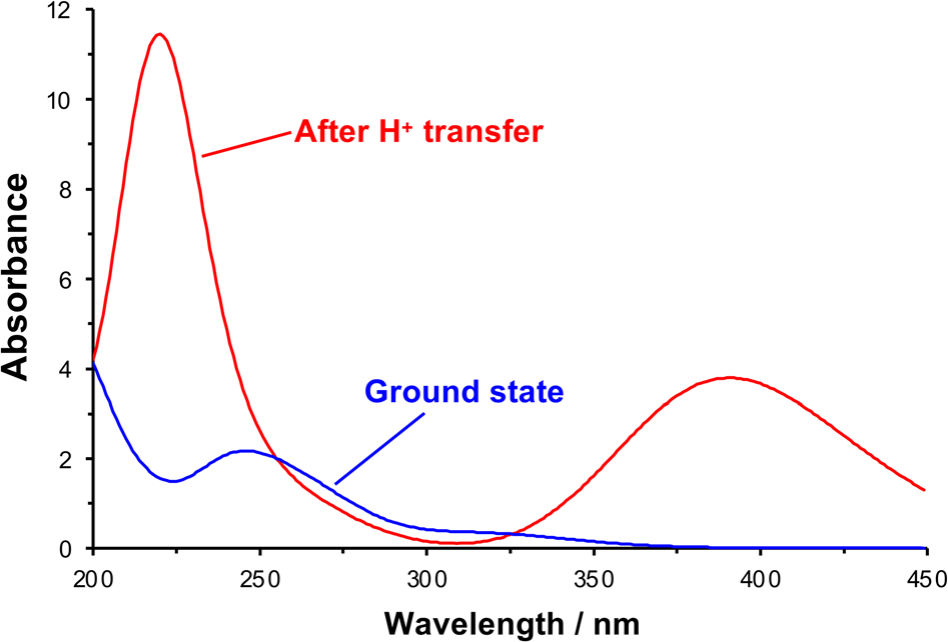
Theoretical UV-visible spectra of initial and proton transfer conformations along photo-deprotection mechanism.

After initial excitation by absorption of a photon, there is a change in the conformation of the NO_2_ group; in the ground state it is planar, but it is no longer planar in the excited state, moving towards a pyramidal conformation (the ONO bond angle changes from 124.3° to 105.4°). In addition, there is a small lengthening of the N–O bonds, suggesting a change in local electronic configuration (ESI†). The absorption spectrum was calculated after formation of the acid-nitro intermediate (Fig. 6). The literature reports a substantial red-shift in the position of the π → π* peak for the intermediate, something that is clearly replicated in the calculated spectrum. In the calculated spectrum, the π → π* transition is seen at 390 nm with an oscillator strength of 0.178, compared to 320 nm (0.015) for the ground state.

The molecular orbitals of the initial and post-proton transfer forms of the nitrophenyl protecting group were calculated (Fig. 5b, c, e and f). In the ground state, the highest occupied molecular orbital (HOMO) is localized predominantly over the carbamate section, while the lowest unoccupied molecular orbital (LUMO) is found over the nitrophenyl. Thus, the HOMO–LUMO transition would necessitate intramolecular charge transfer (ICT) with an associated low oscillator strength. Instead, as the transition density shows, the π → π* transition is entirely localized on the nitro-group in line with the change in geometry after optimization and involves deeper-lying orbitals. However, both the HOMO and LUMO are localized across the nitrophenyl group in the acid-nitro species, explaining the observed red shift in the π → π* transition. The difference in energy between the initial NPPOC molecule and the final products is merely 100 kJ mol^−1^. Thus, the main energetic barrier during photo-deprotection is the π → π* transition, which leads to abstraction of a proton by the nitro group.

**Fig. 7:**
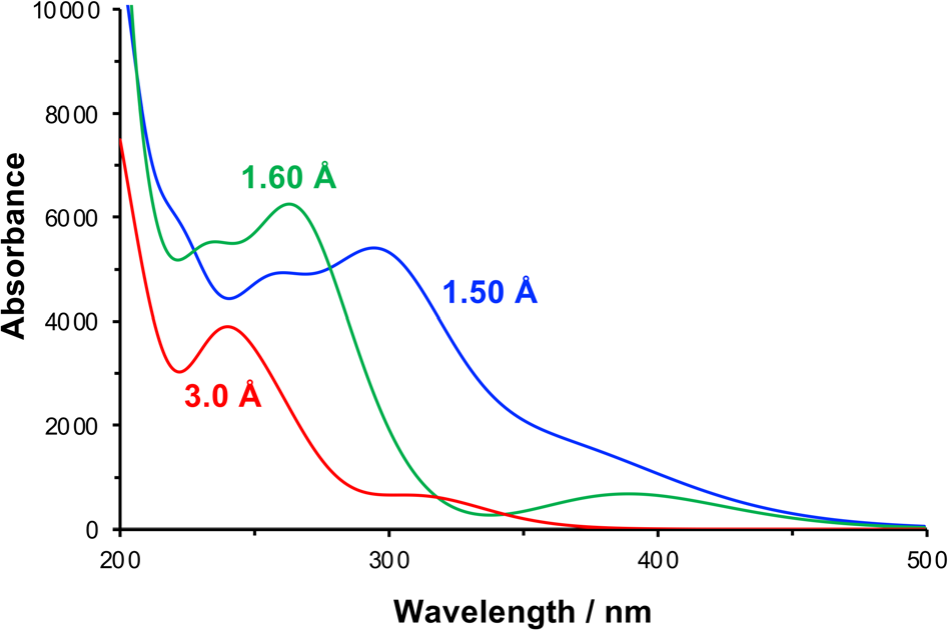
Calculated absorption spectra for NPPOC in the unstrained equilibrium (N–N separation 3.0 Å) and in compressed conformations (N–N separations 1.6 and 1.5 Å).

The effect of compression of the nitrophenyl group, as might be expected in the region of contact between the monolayer and the AFM probe in the lithographic experiment, was examined using DFT. The separation between the nitro and carbamate nitrogen atoms was varied systematically from its unstrained equilibrium value of 3.0 Å. For each N–N separation the structure was optimized and the lowest energy conformation was determined. Spectra were calculated for the equilibrium and the strained configurations (Fig. 7).

At a separation of 1.5 Å, a substantial red shift is observed in the π → π* transition. The total energy of the compressed conformation is higher than that of the equilibrium conformation, but within the range of energies that can be accessed by the loads applied here. Under these conditions the electron density is distributed differently. In contrast to the unstrained conformation, the HOMO is now positioned over the nitro group (Fig. 5f), while the LUMO is also shared across the carbamate group (Fig. 5g). The π → π* transition is therefore the HOMO–LUMO transition and its energy is found to be greatly reduced. The corresponding transition density indeed shows that the π → π* transition is in this case an ICT transition, moving charge density from the carbamate group onto the nitro group.

For some configurations, the reduction was such that the energy barrier was as small as 1.2 eV, equivalent to light with a wavelength of *ca.* 1000 nm, *i.e.* in the near infra-red region of the spectrum rather than the near-UV region. We cannot say what the precise conformation of the nitrophenyl-protected silanes is in the contact region during the lithographic process or whether these are instantaneous or geometry-relaxed conformations. However, the existence of a significant number of configurations in which the π → π* energy is greatly reduced provides strong support for the hypothesis that compression of the nitrophenyl protecting group leading to a change in the energy barrier to deprotection is the main mechanism for the lithographic process described here. We hypothesize that scattered photons from the red laser used to measure the cantilever deflection in a commercial AFM system possess sufficient energy to cause photo-deprotection of the strained nitrophenyl protecting groups, and would account for the facile, well-controlled deprotection demonstrated in Fig. 1–4.

## Conclusions

Using an AFM probe at loads too small to cause mechanical wear it is possible to write nanolines in films of aminosilanes with photoremovable, protein-resistant nitrophenyl protecting groups. Line widths in the range 20–50 nm can be achieved repeatably. Nanolines are readily derivatized by reaction with glutaraldehyde and aminobuytyl nitrilo triacetic acid, enabling complexation with Ni^2+^ and site-specific binding of His-tagged green fluorescent protein. The lithographic process is attributed to a reduction in the π → π* energy in the nitrophenyl group, from 3.1 eV to as little as ∼1.2 eV, enabling deprotection by ambient scattered light (for example, scattered light from the laser used to measure the cantilever deflection). This enables a type of lens-less photolithography with a resolution far better than the diffraction limit at writing rates that may be as high as 100 mm s^−1^.

## Data availability

Data for this paper, including spreadsheets used to generate Fig. 1–4, 6 and 7 are available at the University of Sheffield's Online Data Archive (ORDA) at 10.15131/shef.data.21842718. Information on the calculations leading to Fig. 5 is provided in the ESI† file.

## Author contributions

R. E. D. and O. S. B. carried out nanolithography. O. S. B. and A. J. H. M. M. performed DFT calculations. G. J. L. conceived and designed the work and drafted the manuscript.

## Conflicts of interest

There are no conflicts to declare.

## Supplementary Material

SC-014-D2SC06305K-s001
