# Supplementary material for: Tribochemical nanolithography: selective mechanochemical removal of photocleavable nitrophenyl protecting groups with 23 nm resolution at speeds of up to 1 mm s−1
Source: Chem Sci. 2023 Jan 16;14(7):1752–61. doi: 10.1039/d2sc06305k (PMC9931061; doi:10.1039/d2sc06305k)
Supplement: SC-014-D2SC06305K-s001 [file SC-014-D2SC06305K-s001.pdf]

Electronic Supplementary Material (ESI) for Chemical Science.
This journal is © The Royal Society of Chemistry 2023

Tribochemical Nanolithography: Photolithography without Optics
Supplementary information

Robert E. Ducker,[1] Oscar Siles Brügge,[1,2] Anthony J. H. M. Meijer,[1] and Graham J. Leggett[1]

[1]*Department of Chemistry, University of Sheffield, Sheffield, S3 7HF, UK*
[2]*Department of Chemistry, University of Nottingham, University Park, Nottingham, NG7 2RD, UK*

(Dated: October 25, 2022)

CONTENTS

S1. Methods S3
S1.1. Surface Preparation S3
S1.2. Tribochemical Nanolithography S3
S1.3. Surface Functionalization S4
S1.4. Density Functional Theory S4

S2. Lithographic Process S5

S3. Density Functional Theory S6

S4. Calculations on NPPOC (folded form; S_0) S9
S4.1. Cartesian Co-ordinates (XYZ format) S9
S4.2. Frequencies S11

S5. Calculations on NPPOC (straight form; S_0) S13
S5.1. Cartesian Co-ordinates (XYZ format) S13
S5.2. Frequencies S15

S6. Calculations on NPPOC (folded form; S_1) S17
S6.1. Cartesian Co-ordinates (XYZ format) S17

S7. Calculations on NPPOC (H-transfer; relaxed; S_1) S19
S7.1. Cartesian Co-ordinates (XYZ format) S19

S8. Calculations on NPPOC (H-transfer; relaxed; S_0) S21
S8.1. Cartesian Co-ordinates (XYZ format) S21
S8.2. Frequencies S23

S9. Calculations on NPPOC (after loss of H^+; S_0) S25
S9.1. Cartesian Co-ordinates (XYZ format) S25
S9.2. Frequencies S27

S10. Calculations on NPPOC (Dissociated; S_0) S29
S10.1. Cartesian Co-ordinates (XYZ format) S29

S1. METHODS

S1.1. Surface Preparation

(Methoxyheptaethyleneglycol)nitrophenylethoxycarbonyl-protected aminopropyl(triethoxy silane) (OEG-NPEOC-APTES) was synthesized by AF ChemPharm Ltd (Sheffield, UK). {*N*-[2-(2-Nitrophenyl)propan-1-oxycarbonyl]-3-aminopropyl}-triethoxysilane (NPPOC-APTES) was synthesized according to our previously reported methodology.[1] Glass slides (Menzel-Glaser, Braunschweig, Germany) and silicon wafers (PI-KEM Ltd, Tamworth, UK) were used as substrates. A thermal oxide was grown on the silicon wafers by heating them in a furnace for 6 h at 950 °C and allowing them to slowly cool to room temperature. Prior to use, all substrates were cleaned in piranha solution, a 1:3 mixture of hydrogen peroxide and sulfuric acid (***Caution! Piranha solution is an extremely strong oxidizing agent and can detonate upon contact with organic materials***) and rinsed thoroughly in deionized water (Veolia Water Technologies, High Wycombe, UK). The substrates were placed in an RCAII bath (hydrogen peroxide, ammonia, and water in the ratio 1:2:5) for 20 min, allowed to cool and rinsed thoroughly with deionized water. The substrates were dried at 120 °C for 2 h. Films of adsorbed silanes were formed by immersing clean, dry substrates in a solution of the silane in toluene (30 μL in 30 mL) for 48 h. The substrates were removed from the silane solution and washed in toluene, then a 50:50 (vol/vol) mix of toluene and ethanol, and finally with pure ethanol. The substrates were annealed at 120 °C for 20 minutes under vacuum. Samples were stored sealed in the dark until needed and used within one month of preparation.

S1.2. Tribochemical Nanolithography

Tribochemical removal of nitrophenyl protecting groups was performed using a Nanoscope IIIa with nanolithography module. Tapping mode AFM probes (OTESPA-R3, Bruker, Karlsruhe, Germany) with nominal spring constants of 26 N m^{-1} and a tip radius of 7-10 nm were used. The spring constant k was measured for each cantilever using the thermal tune method, and the deflection sensitivity σ was determined by bringing the probe into contact with a mica surface. Nanolithography was performed in contact mode. The applied load normal to the surface F_N was calculated using the following equation:

$$F_{\mathrm{N}} = k\sigma \times \text{setpoint} \quad (1)$$

After nanolithography, the substrates were washed with ethanol or deionized water and dried under a stream of nitrogen.

S1.3 Surface Functionalization

To enable attachment of green fluorescent protein (GFP) to amine patterns formed by nanolithography, samples were first placed in a 25% (v/v) glutaraldehyde solution (pH 5) for 1 h in order to form an aldehyde-functionalized surface. Subsequently, the samples were immersed in a 10 mM aqueous solution of N-(5-amino-1-carboxypentyl)iminodiacetic acid (ABNTA) at pH 5 for 18 h to produce NTA functional surfaces. Ni^{2+}-chelated surfaces were prepared by immersing the samples in 100 mM $NiCl_2$ for 4 h, and complexed to His-GFP by immersion in a solution of the protein in buffer for 18 h. To remove the His-tagged GFP from the surface, samples were immersed in imadazole (100 mM, pH 7.4) solution for 4 h, and then rinsed with ammonium acetate and dried gently with a stream of nitrogen.

S1.4. Density Functional Theory

The ground-state geometry of a simpler NPPOC molecule (without oligoethylene glycol sidechain, and with a methyl replacing the APTES group) in the gas-phase and in water was calculated for a number of different functionals and basis sets using Gaussian 09. From excited-state energy calculations, theoretical UV-Vis spectra were obtained and compared to the experimental value obtained in acetonitrile. The functional and basis set that resulted in spectra that most closely resembled empirical data were selected for use in further calculations.

The ground-state geometries for a variety of different conformations along the proposed NPEOC deprotection pathway were optimized. Additionally, geometries as various bonds in NPEOC were stretched by scanning across a range of separation values were also obtained. The resulting ground-state energy vs. bond length plots were fitted with a Morse oscillator potential to obtain a relative measure of the bond-dissociation energy.

Calculations in the excited-state geometry optimizations were performed using the "Optimization" job type and "TD-SCF" method, with 6 excited-state levels being investigated to arrive at a final geometry for the n = 2 energy level (N = 6, with state of interest root N = 1). UV-Vis spectra (and associated energy level transitions) were calculated using the "Energy" job type and "TD-SCF" method for 100 excited-state energy levels (N = 100), with resulting data exported into CSV files.

Results given above are for simulations run *in vacuo*. However, simulations run in a water environment were also performed using the integral equation formulism polarizable continuum model (IEFPCM), developed by Tomasi *et al.*[2] and Pascual-Ahuir *et al.*[3] These give similar results.

S2. LITHOGRAPHIC PROCESS

To test whether pattern formation occurred via mechanical wear of the surface, behaviour was compared for APTES films at loads of 500 nN and 15 N (Figure S1). For these silanes that do not have a photoremovable protecting group, patterning was not observed at a load of 500 nN (the load typically selected in the lithographic experiments described in the main manuscript). However, modification of APTES films was observed on increasing the load above 12.5 μN, suggesting that there was a very high load threshold for mechanical wear. Thus, the patterning process observed for OEG-NPEOC-APTES films at lower loads is not attributable to mechanical wear. In contrast, feature formation is observed at loads of 500 nN for OEG-NPEOC-APTES films. Moreover, modification of APTES films at high loads leads to the accumulation of debris along the edge of the patterned structures (bright features in Figure S1(b)), indicative of tip-induced wear. In contrast, such accumulations of debris are not observed for OEG-NPEOC-APTES films even at a load of 15 μN, suggesting a different mechanism of surface modification.

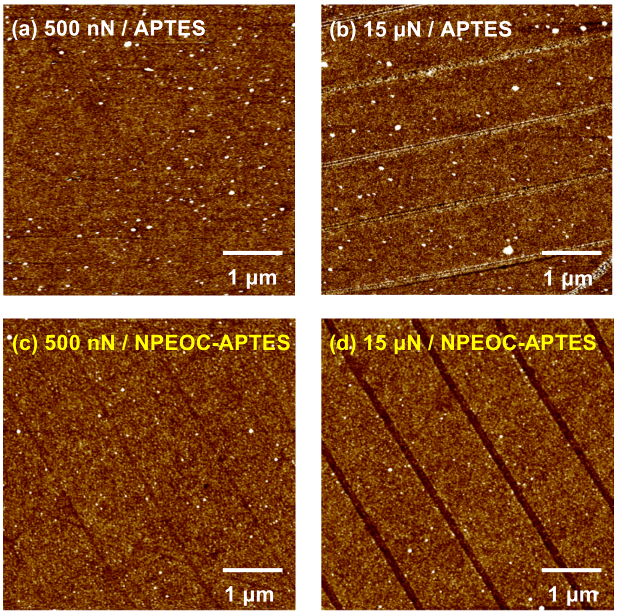

***Figure S1**: Tapping mode images showing the effect of the load on tip-induced modification of siloxane films. Top: APTES. Bottom: OEG-NPEOC-APTES.*

S3. DENSITY FUNCTIONAL THEORY

A number of density functionals and basis sets were compared, by testing their ability to reproduce the absorption spectrum on the NPPOC functional group. The ω-B97XD functional[4] was selected. Figure S2 shows the experimental spectrum for NPEOC-APTES (black) together with calculated spectra produced using the ω-B97XD functional in conjunction with three different basis sets. The 6-311++G(d,p) was judged to be the best. This basis set includes a diffuse function in order to better represent orbitals further from the nucleus, including those of lighter atoms.

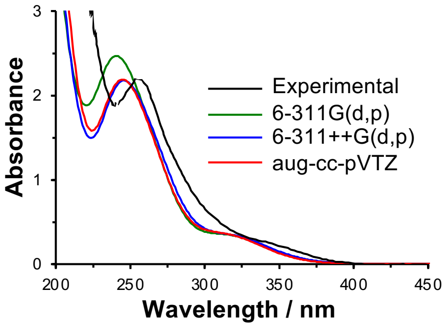

***Figure S2**: Absorption spectra for NPPOC calculated using the ω-B97XD functional in conjunction with three different basis sets.*

Two optimised ground-state configurations were determined from the DFT calculations (Supporting Information), described as "straight" and "folded". The folded

conformation had slightly lower energy than the "straight" conformation and was thus used as the starting conformation for subsequent calculations.

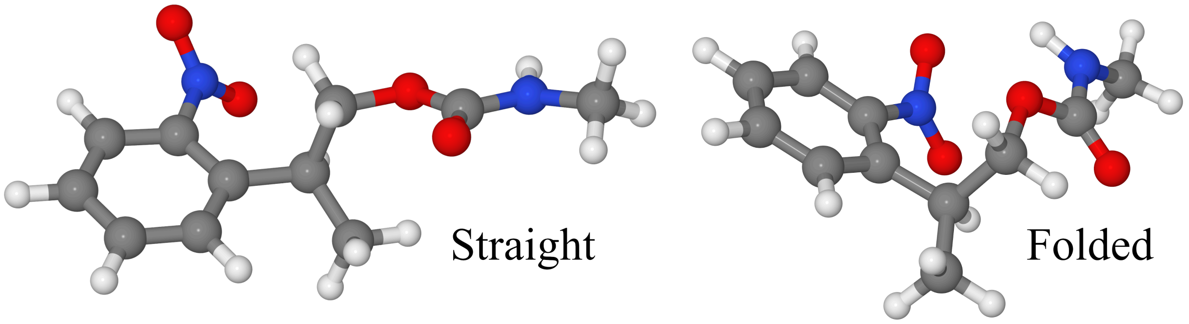

Figure S3: *Straight and folded conformations for NPPOC determined from DFT calculations.*

After initial excitation by absorption of a photon, there is a change in the conformation of the NO_2 group; in the ground state it is planar, but it is no longer planar in the excited state, moving towards a pyramidal conformation (the ONO bond angle changes from 124.3º to 105.4º), and there is a small lengthening of the N–O bonds, suggesting a change in local electronic configuration (Figure S4).

The absorption spectrum was calculated after formation of the aci-nitro intermediate (Figure S5). The literature reports a substantial red-shift in the position of the $\pi \rightarrow \pi^*$ peak for the intermediate, something that is clearly replicated in the calculated spectrum. In the calculated spectrum, the $\pi \rightarrow \pi^*$ transition is seen at 390 nm with an oscillator strength of 0.178, compared to 320 nm (0.015) for the ground state. This $S_0 \rightarrow S_1$ transition has a strength an order of magnitude greater than in the initial ground-state conformation, albeit at a lower energy, but is unlikely to be a factor in subsequent steps in the decomposition of the molecule.

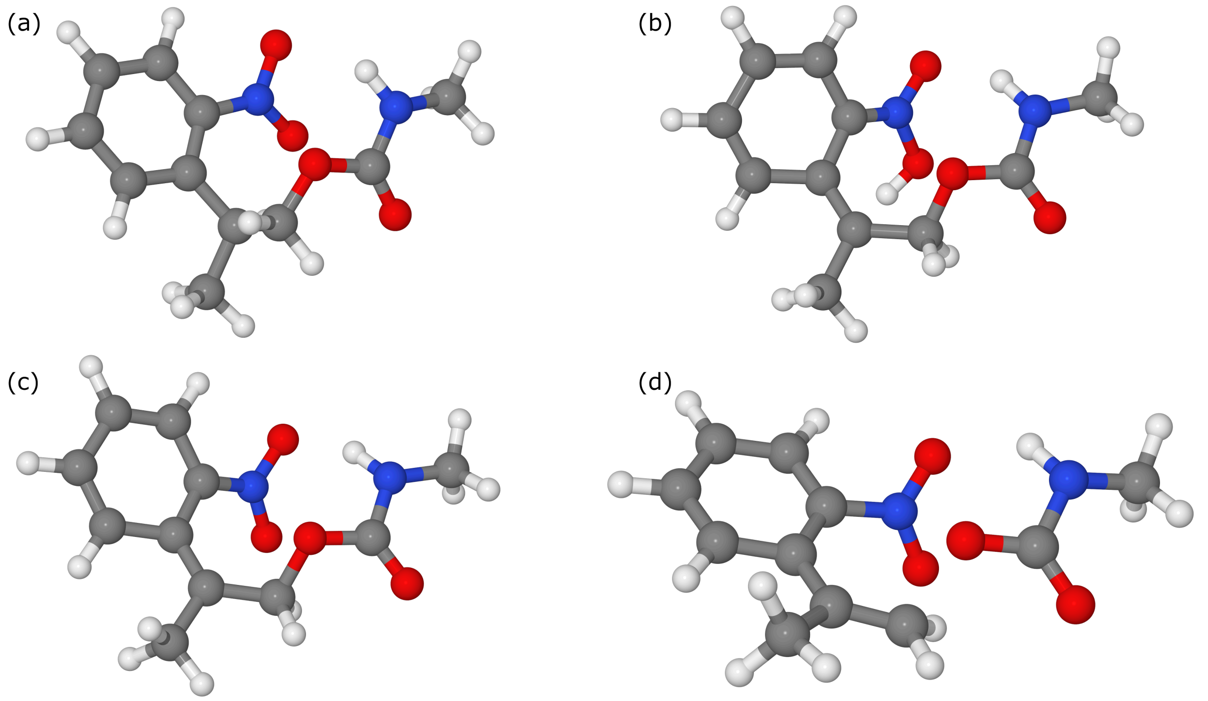

Figure S4: Optimized geometries at different steps along the proposed photo-deprotection pathway. (a) After irradiation with UV light. (b) After proton transfer and relaxation (intermediate formation). (c) After proton loss (ionic form). (d) After decomposition of intermediate.

1 Alang-Ahmad, S. A. *et al.* Micrometer- and Nanometer-Scale Photopatterning Using 2-Nitrophenylpropyloxycarbonyl-Protected Aminosiloxane Monolayers. *J. Am Chem. Soc.* **131**, 1513–1522 (2009).

2 Miertuš, S., Scrocco, E. & Tomasi, J. Electrostatic interaction of a solute with a continuum. A direct utilizaion of AB initio molecular potentials for the prevision of solvent effects. *Chemical Physics* **55**, 117-129, doi:https://doi.org/10.1016/0301-0104(81)85090-2 (1981).

3 Pascual-ahuir, J. L., Silla, E. & Tuñon, I. GEPOL: An improved description of molecular surfaces. III. A new algorithm for the computation of a solvent-excluding surface. *Journal of Computational Chemistry* **15**, 1127-1138, doi:https://doi.org/10.1002/jcc.540151009 (1994).

4 Mardirossian, N. & Head-Gordon, M. ωB97X-V: A 10-parameter, range-separated hybrid, generalized gradient approximation density functional with nonlocal correlation, designed by a survival-of-the-fittest strategy. *Physical Chemistry Chemical Physics* **16**, 9904-9924, doi:10.1039/C3CP54374A (2014).

S4. CALCULATIONS ON NPPOC (FOLDED FORM; S_0)

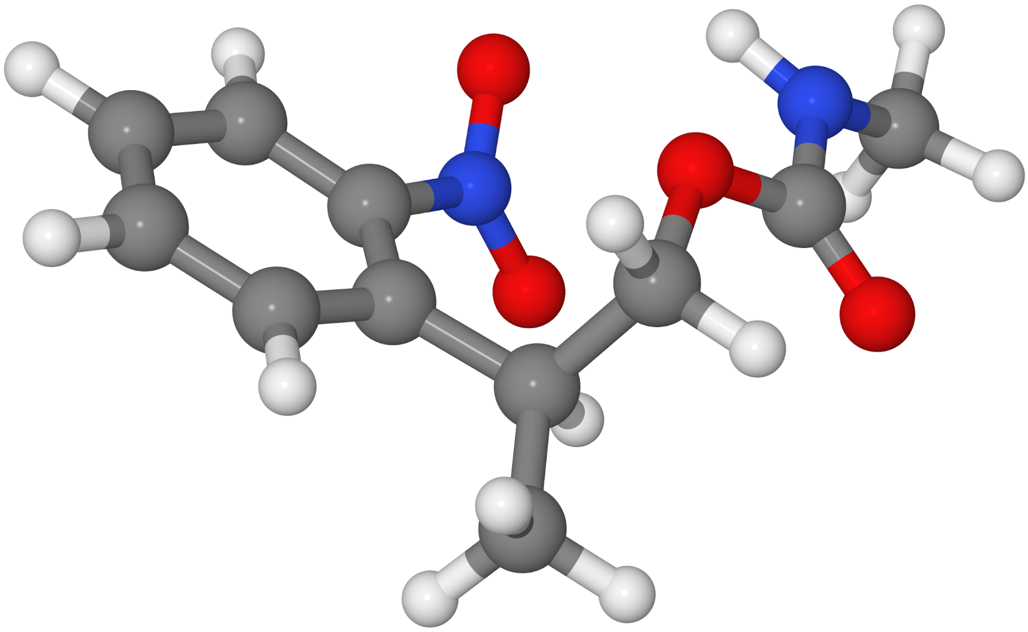

Route	: # opt freq wb97xd/6-311++g(d,p) geom=connectivity int=ultrafine		
SMILES	: CC(COC(=O)NC)c1ccccc1N(=O)=O		
Formula	: $C_{11}H_{14}N_2O_4$		
Charge	: 0		
Multiplicity	: 1		
Dipole	: 11.4787	Debye	
Energy	: -837.89556198	a.u.	
Gibbs Energy	: -837.68941400	a.u.	
Number of imaginary frequencies	: 0		

S4.1. Cartesian Co-ordinates (XYZ format)

31

C	3.83181906	-0.11519900	0.50298500
C	2.86214900	0.82782698	0.19079000
C	1.56898999	0.47289801	-0.20184501
C	1.31238794	-0.90280700	-0.24029800
C	2.25747204	-1.86417699	0.09128400
C	3.53369808	-1.46973300	0.45401001
H	4.82389688	0.21398801	0.79010600
H	3.12104988	1.87700295	0.25349799
H	1.98114705	-2.90989399	0.05584300
H	4.28265715	-2.21307397	0.69794202
N	0.00264400	-1.44217300	-0.64833099
O	-0.43630999	-2.37853289	-0.00402400
O	-0.55119902	-0.95740098	-1.61332595
C	0.51871902	1.54634500	-0.44354999
H	-0.14104100	1.21987998	-1.24654305

C	1.09878194	2.90407491	-0.84441602
H	1.80952501	2.80593491	-1.66769195
H	0.28789699	3.55715895	-1.17307103
H	1.60404396	3.40451598	-0.01243000
C	-0.34633601	1.72408998	0.81304598
H	-0.99615401	2.59180188	0.69191200
H	0.27970201	1.85467696	1.69842899
O	-1.13978004	0.56979501	1.06660295
C	-2.36596704	0.55329603	0.46953499
O	-2.84265208	1.49803901	-0.11510200
N	-2.95404005	-0.64915800	0.65510899
H	-2.34631705	-1.39757502	0.95099998
C	-4.15505219	-0.99264401	-0.07910500
H	-3.93546295	-1.24915302	-1.12033999
H	-4.63600206	-1.84178305	0.40711600
H	-4.83806181	-0.14406800	-0.06510400

S4.2. Frequencies

Mode	IR frequency	IR intensity	Raman intensity
1	27.94860000	0.17240000	0.00000000
2	42.56210000	1.64540000	0.00000000
3	71.68430000	1.74060000	0.00000000
4	79.18040000	0.56160000	0.00000000
5	88.10130000	7.10640000	0.00000000
6	98.84240000	3.30950000	0.00000000
7	108.33450000	0.97500000	0.00000000
8	148.46310000	0.85210000	0.00000000
9	177.04150000	2.27230000	0.00000000
10	184.72260000	2.37980000	0.00000000
11	226.75680000	6.06850000	0.00000000
12	260.02090000	1.55880000	0.00000000
13	265.11070000	2.79330000	0.00000000
14	275.88310000	1.56270000	0.00000000
15	310.31140000	1.45510000	0.00000000
16	370.03070000	32.09800000	0.00000000
17	383.66750000	0.94550000	0.00000000
18	396.13320000	3.44380000	0.00000000
19	407.88650000	2.09700000	0.00000000
20	446.68800000	0.08990000	0.00000000
21	555.82240000	3.12770000	0.00000000
22	574.63000000	73.46850000	0.00000000
23	583.79260000	48.02510000	0.00000000
24	591.72020000	45.59370000	0.00000000
25	609.48530000	0.37260000	0.00000000
26	685.82020000	6.70730000	0.00000000
27	713.88210000	27.10290000	0.00000000
28	741.63500000	23.42450000	0.00000000
29	773.22490000	21.92860000	0.00000000
30	783.80940000	15.50480000	0.00000000
31	795.80940000	19.07210000	0.00000000
32	821.09170000	11.50780000	0.00000000
33	891.31790000	20.46240000	0.00000000
34	899.08910000	9.59700000	0.00000000
35	915.13120000	14.73380000	0.00000000
36	930.70270000	8.39670000	0.00000000
37	983.12320000	12.81110000	0.00000000
38	989.17240000	0.87080000	0.00000000
39	1023.09930000	0.21240000	0.00000000
40	1058.27150000	34.56950000	0.00000000
41	1068.51180000	12.16630000	0.00000000
42	1083.15620000	7.64750000	0.00000000
43	1103.62840000	1.14500000	0.00000000
44	1132.16300000	0.76120000	0.00000000
45	1152.12730000	21.14890000	0.00000000
46	1154.39080000	13.55950000	0.00000000
47	1183.12700000	149.66170000	0.00000000
48	1188.60240000	2.15160000	0.00000000
49	1200.25120000	2.86540000	0.00000000
50	1202.68720000	13.46800000	0.00000000
51	1233.38580000	8.45990000	0.00000000
52	1282.17500000	254.29580000	0.00000000
53	1293.66620000	33.12950000	0.00000000
54	1321.12470000	10.99350000	0.00000000
55	1340.59980000	14.84970000	0.00000000
56	1355.47040000	7.35850000	0.00000000
57	1389.55040000	1.30040000	0.00000000
58	1417.71700000	8.38960000	0.00000000
59	1423.82750000	5.79300000	0.00000000
60	1458.28990000	225.30500000	0.00000000

61	1461.18690000	10.61920000	0.00000000
62	1480.70650000	10.62910000	0.00000000
63	1490.08260000	1.64250000	0.00000000
64	1494.46620000	27.85810000	0.00000000
65	1506.55320000	9.52740000	0.00000000
66	1509.26360000	9.00900000	0.00000000
67	1513.82720000	17.79830000	0.00000000
68	1534.52290000	9.51580000	0.00000000
69	1584.56300000	306.49960000	0.00000000
70	1643.64820000	106.41690000	0.00000000
71	1657.16860000	92.96190000	0.00000000
72	1690.51860000	48.38410000	0.00000000
73	1830.67010000	366.01790000	0.00000000
74	3045.04900000	34.66670000	0.00000000
75	3045.34700000	33.13870000	0.00000000
76	3080.34090000	31.88380000	0.00000000
77	3114.88890000	16.13210000	0.00000000
78	3125.55300000	23.72710000	0.00000000
79	3137.03880000	4.13920000	0.00000000
80	3138.17310000	11.18010000	0.00000000
81	3149.92700000	9.91630000	0.00000000
82	3163.02960000	7.91250000	0.00000000
83	3197.88510000	2.38720000	0.00000000
84	3215.27980000	6.97830000	0.00000000
85	3225.93940000	5.07750000	0.00000000
86	3233.74030000	3.00660000	0.00000000
87	3654.82090000	50.94170000	0.00000000

S5. CALCULATIONS ON NPPOC (STRAIGHT FORM; S_0)

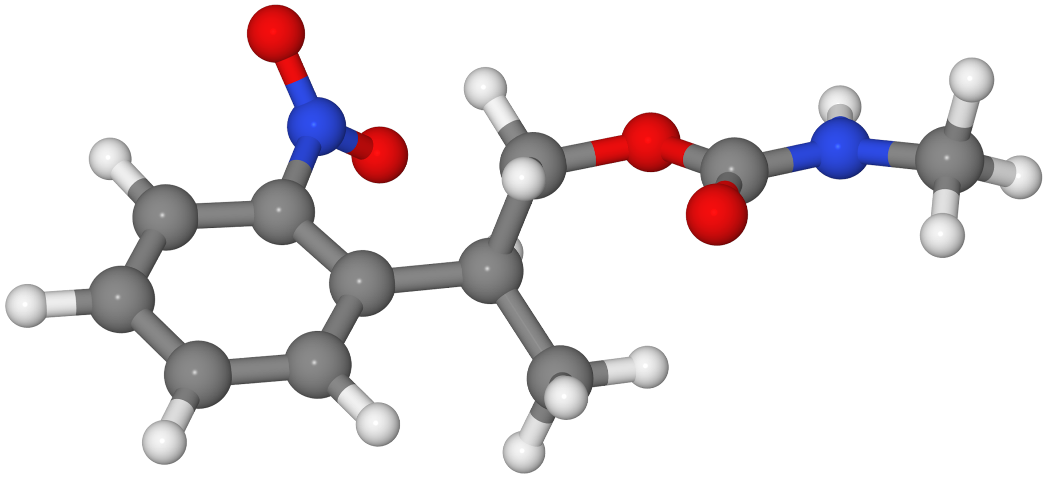

Route	: # opt freq wb97xd/6-311++g(d,p) geom=connectivity int=ultrafine	
SMILES	: CC(COC(=O)NC)c1ccccc1N(=O)=O	
Formula	: $C_{11}H_{14}N_2O_4$	
Charge	: 0	
Multiplicity	: 1	
Dipole	: 5.9868	Debye
Energy	: -837.89045085	a.u.
Gibbs Energy	: -837.68782300	a.u.
Number of imaginary frequencies	: 0	

S5.1. Cartesian Co-ordinates (XYZ format)

31

C	-2.61938095	-1.74748397	0.07618600
C	-2.31463695	-0.39372799	0.13432001
C	-1.07577205	0.07062900	0.58009601
C	-0.15383001	-0.91917700	0.93807101
C	-0.42469299	-2.27741790	0.86495101
C	-1.67565596	-2.69671702	0.44329801
H	-3.59964395	-2.05931497	-0.26543799
H	-3.06278491	0.32448399	-0.17520501
H	0.34448999	-2.98723412	1.14114904
H	-1.90545702	-3.75415111	0.39695400
N	1.19797599	-0.57461500	1.42426002
O	2.12914705	-1.19592297	0.95130098
O	1.30101001	0.28093201	2.28110504
C	-0.74066401	1.55359399	0.56174499
H	-0.13085701	1.77529097	1.43737698
C	-1.96510196	2.46817803	0.59816098
H	-2.61973310	2.20991707	1.43391001
H	-1.64273095	3.50198102	0.73601401
H	-2.53862810	2.43202710	-0.33103400
C	0.12037600	1.81742704	-0.68252403
H	-0.44903600	1.61653495	-1.59259605

H	1.01877296	1.19703603	-0.67646801
O	0.58396500	3.16604209	-0.69946802
C	-0.02664200	4.02552795	-1.55026197
O	-0.90846598	3.72469211	-2.32130790
N	0.48873100	5.26752806	-1.39170003
H	1.28928900	5.35468388	-0.78905100
C	0.11919900	6.35918379	-2.26666689
H	0.71900499	6.37603092	-3.18245602
H	-0.92883199	6.25047302	-2.54367304
H	0.24852200	7.30421209	-1.73761594

S5.2. Frequencies

Mode	IR frequency	IR intensity	Raman intensity
1	21.84030000	3.37850000	0.00000000
2	28.83980000	1.45840000	0.00000000
3	38.04540000	1.13610000	0.00000000
4	45.83770000	6.60500000	0.00000000
5	54.83760000	0.43150000	0.00000000
6	66.53480000	1.24860000	0.00000000
7	93.97510000	0.57640000	0.00000000
8	132.01610000	1.10670000	0.00000000
9	134.24110000	6.91460000	0.00000000
10	184.73120000	1.05230000	0.00000000
11	229.54010000	3.04640000	0.00000000
12	255.36600000	3.09930000	0.00000000
13	269.58970000	2.76400000	0.00000000
14	284.67070000	2.37570000	0.00000000
15	300.24250000	0.69700000	0.00000000
16	342.43280000	5.52520000	0.00000000
17	378.93360000	24.35610000	0.00000000
18	391.37630000	2.49310000	0.00000000
19	411.98610000	1.71940000	0.00000000
20	468.03410000	29.92010000	0.00000000
21	480.03820000	61.33070000	0.00000000
22	525.69580000	10.44340000	0.00000000
23	559.12390000	3.89380000	0.00000000
24	598.60370000	1.35920000	0.00000000
25	636.84920000	0.23310000	0.00000000
26	695.70240000	7.10290000	0.00000000
27	707.16250000	6.99540000	0.00000000
28	743.51630000	17.79170000	0.00000000
29	768.18730000	35.96230000	0.00000000
30	791.99330000	10.27730000	0.00000000
31	805.70100000	19.07400000	0.00000000
32	819.83840000	6.30230000	0.00000000
33	886.23360000	22.42240000	0.00000000
34	898.42900000	8.55250000	0.00000000
35	905.74920000	1.10240000	0.00000000
36	926.71910000	9.20050000	0.00000000
37	966.30550000	13.23180000	0.00000000
38	988.01360000	1.74650000	0.00000000
39	1023.69860000	0.40260000	0.00000000
40	1048.23370000	85.92470000	0.00000000
41	1059.22000000	9.28280000	0.00000000
42	1087.85760000	2.41590000	0.00000000
43	1101.78500000	4.03980000	0.00000000
44	1143.32400000	1.42930000	0.00000000
45	1155.67220000	23.73500000	0.00000000
46	1164.04340000	23.67300000	0.00000000
47	1188.28930000	10.83320000	0.00000000
48	1193.87830000	28.87340000	0.00000000
49	1202.67870000	3.98110000	0.00000000
50	1207.54650000	111.66150000	0.00000000
51	1234.92740000	3.61990000	0.00000000
52	1273.91080000	455.46820000	0.00000000
53	1302.92460000	8.80990000	0.00000000
54	1324.07430000	9.01850000	0.00000000
55	1340.88200000	19.11960000	0.00000000
56	1354.59420000	8.39830000	0.00000000
57	1402.14020000	9.74580000	0.00000000
58	1417.39200000	4.17070000	0.00000000
59	1435.47710000	2.86330000	0.00000000
60	1458.10430000	173.49480000	0.00000000

61	1461.32380000	18.45100000	0.00000000
62	1489.14930000	3.05500000	0.00000000
63	1492.85290000	2.25720000	0.00000000
64	1497.05260000	34.80310000	0.00000000
65	1501.55680000	15.69550000	0.00000000
66	1514.87210000	42.34220000	0.00000000
67	1517.40630000	7.12280000	0.00000000
68	1535.73460000	9.01710000	0.00000000
69	1581.62390000	433.23940000	0.00000000
70	1647.12220000	92.74980000	0.00000000
71	1658.87700000	169.26890000	0.00000000
72	1690.91970000	73.91400000	0.00000000
73	1826.31300000	335.74120000	0.00000000
74	3042.54960000	65.14380000	0.00000000
75	3052.91680000	15.13400000	0.00000000
76	3080.15300000	27.14950000	0.00000000
77	3120.64770000	22.40780000	0.00000000
78	3121.24650000	5.27250000	0.00000000
79	3133.94130000	18.52720000	0.00000000
80	3135.93550000	15.09800000	0.00000000
81	3146.15880000	6.67270000	0.00000000
82	3163.00300000	6.46980000	0.00000000
83	3198.68900000	2.15010000	0.00000000
84	3214.87760000	6.80050000	0.00000000
85	3227.73730000	1.99720000	0.00000000
86	3231.72640000	4.08060000	0.00000000
87	3692.58730000	46.57330000	0.00000000

S6. CALCULATIONS ON NPPOC (FOLDED FORM; S_1)

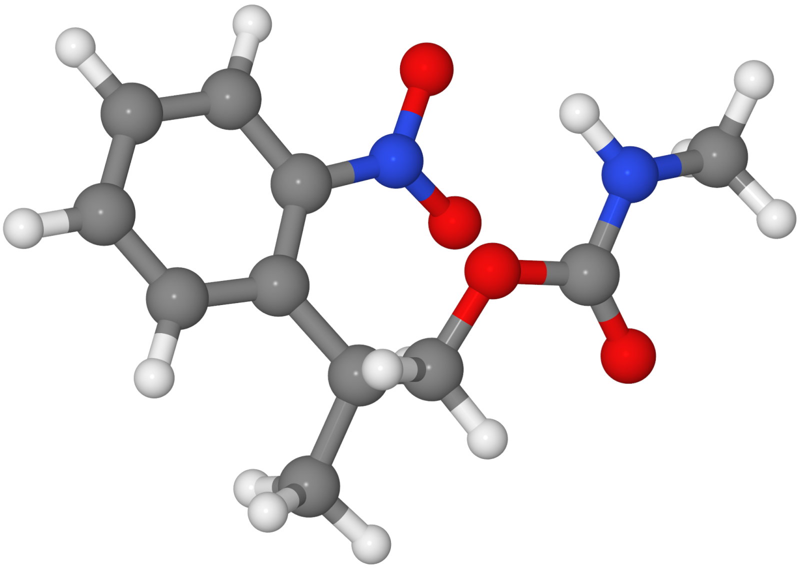

Route	:	# opt=maxstep=3 td=(nstates=6,root=1) wb97xd/6-311++g(d,p) nosymm geom
	:	=connectivity int=ultrafine iop(1/19=15)
SMILES	:	CC(COC(=O)NC)c1ccccc1N(=O)=O
Formula	:	$C_{11}H_{14}N_2O_4$
Charge	:	0
Multiplicity	:	1
Energy	:	-837.77915912 a.u.

S6.1. Cartesian Co-ordinates (XYZ format)

31

C	-3.70653391	-0.17038199	-0.67987102
C	-2.79748011	0.80764902	-0.29493099
C	-1.53932297	0.50033301	0.21765700
C	-1.24501002	-0.87108397	0.34884700
C	-2.14392805	-1.86970103	-0.04307500
C	-3.37474298	-1.51427996	-0.56195301
H	-4.67281818	0.12050100	-1.07469797
H	-3.07489896	1.84820604	-0.41001001
H	-1.86791503	-2.90851212	0.08531500
H	-4.07638216	-2.28468704	-0.85792601
N	-0.02196700	-1.31957901	0.84134400
O	0.38219699	-2.54053497	0.79329401
O	0.76874298	-0.73165298	1.66492796
C	-0.52657002	1.59934402	0.49058300
H	0.14937100	1.28088295	1.28725195
C	-1.15001905	2.92762399	0.92395401
H	-1.85785699	2.78282499	1.74258006
H	-0.36450300	3.60369992	1.26782596
H	-1.67373204	3.42717290	0.10325500
C	0.33583799	1.82919002	-0.75981897
H	1.01836395	2.66357207	-0.59044701
H	-0.29746300	2.04002905	-1.62447500

O	1.08054602	0.66616601	-1.10131502
C	2.30862403	0.55734199	-0.52247399
O	2.84129906	1.44351006	0.10244200
N	2.83560395	-0.66145098	-0.78326601
H	2.18926692	-1.35738397	-1.11811602
C	4.01550007	-1.10988700	-0.07107800
H	3.78075099	-1.40403795	0.95663500
H	4.44751120	-1.95934606	-0.60082000
H	4.74531794	-0.30157101	-0.04566800

S7. CALCULATIONS ON NPPOC (H-TRANSFER; RELAXED; S_1)

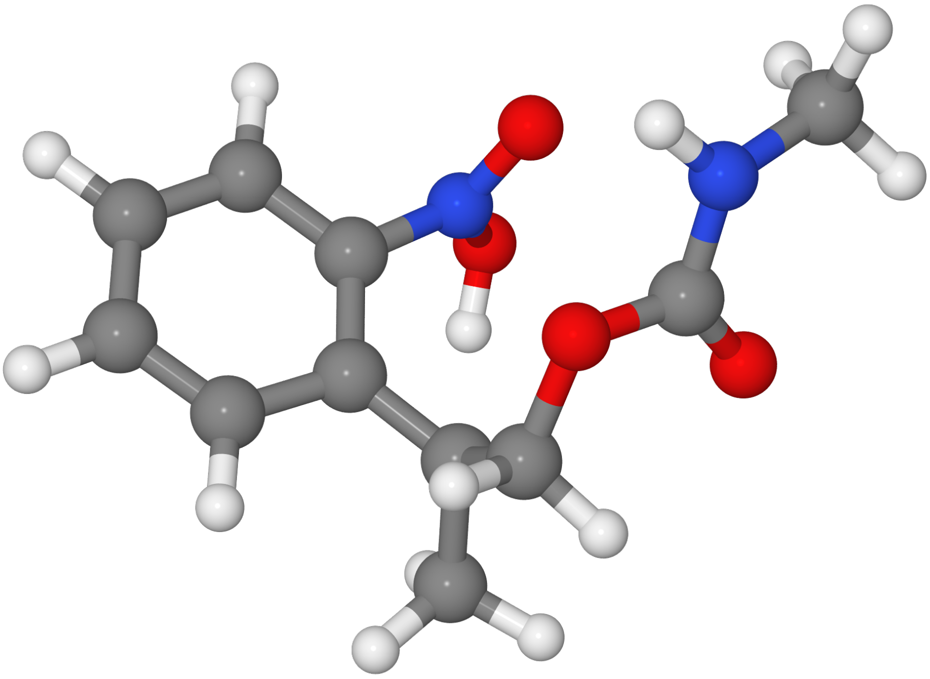

Route : # opt=maxstep=3 td=(nstates=6,root=1) wb97xd/6-311++g(d,p) nosymm geom =connectivity density=current int=ultrafine iop(1/19=15)
SMILES : C[C](COC(=O)NC)c1ccccc1N(O)[O]
Formula : $C_{11}H_{14}N_2O_4$
Charge : 0
Multiplicity : 1
Energy : -837.716697352 a.u.

S7.1. Cartesian Co-ordinates (XYZ format)

```
31

C  -1.37003 -0.24591 -2.92637
C  -0.03523 -0.44910 -2.62879
C   0.43728 -0.69207 -1.32438
C  -0.56868 -0.66476 -0.31780
C  -1.92211 -0.47565 -0.61032
C  -2.32448 -0.26436 -1.91256
H  -1.67580 -0.08411 -3.95403
H   0.69455 -0.45495 -3.43185
H  -2.63567 -0.49645  0.20512
H  -3.37350 -0.11528 -2.14029
N  -0.23976 -0.85202  1.03782
O  -0.32027 -1.92524  1.62380
O   0.25286  0.21942  1.73035
C   1.85139 -0.98134 -1.10815
C   2.81974  0.10549 -1.47077
H   2.54958  1.06492 -1.01759
H   3.83072 -0.14617 -1.13814
H   2.87223  0.28892 -2.56217
```

C	2.31262	-2.37641	-1.36489
H	3.39072	-2.45606	-1.20627
H	2.08749	-2.68792	-2.39878
O	1.65750	-3.39361	-0.58819
C	2.03417	-3.46455	0.70849
O	2.93514	-2.81829	1.19520
N	1.29302	-4.38991	1.36346
H	0.50471	-4.76909	0.86797
C	1.37230	-4.53472	2.80024
H	0.76842	-3.78381	3.31883
H	1.03058	-5.53277	3.07877
H	2.41018	-4.41783	3.11052
H	0.85894	0.66873	1.11947

S8. CALCULATIONS ON NPPOC (H-TRANSFER; RELAXED; S_0)

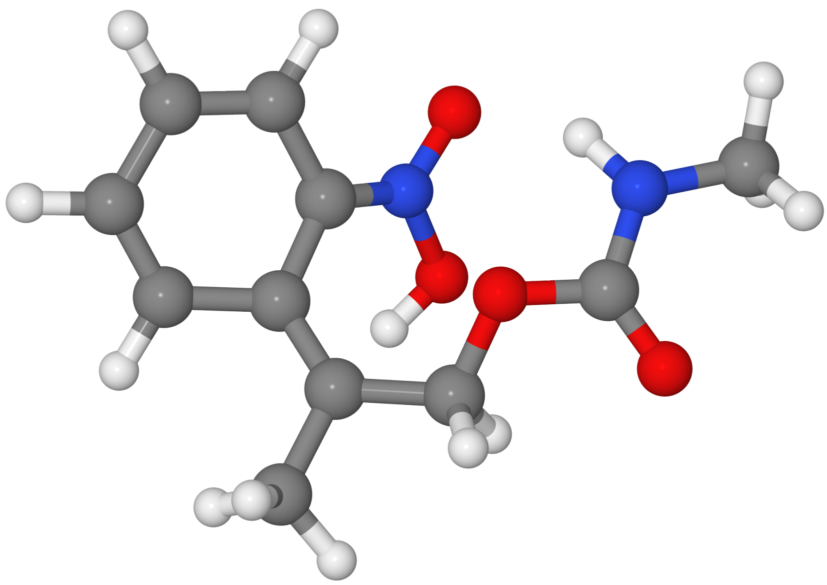

Route	: # opt freq wb97xd/6-311++g(d,p) geom=connectivity int=ultrafine	
SMILES	: C[C](COC(=O)NC)c1ccccc1N(O)[O]	
Formula	: $C_{11}H_{14}N_2O_4$	
Charge	: 0	
Multiplicity	: 1	
Dipole	: 11.4262	Debye
Energy	: -837.82704857	a.u.
Gibbs Energy	: -837.62365900	a.u.
Number of imaginary frequencies	: 0	

S8.1. Cartesian Co-ordinates (XYZ format)

31

C	-1.74821103	0.07536200	-2.60442400
C	-0.64334601	0.75014299	-2.23560095
C	0.29156899	0.18432100	-1.25830996
C	-0.32564399	-0.84674001	-0.40605500
C	-1.39575195	-1.65619397	-0.94354600
C	-2.07927489	-1.20317698	-2.01143003
H	-2.39286089	0.47840601	-3.37705803
H	-0.39095399	1.67766905	-2.73311496
H	-1.66308498	-2.56701303	-0.42517900
H	-2.90983391	-1.77434897	-2.40834999
N	-0.02206800	-1.01659298	0.86772799
O	-0.37459999	-1.94729805	1.59739900
O	0.77788699	-0.08510800	1.51496696
C	1.61843896	0.48779100	-1.30015600
C	2.15111589	1.60698903	-2.15882111
H	1.48134398	2.46618891	-2.19785595
H	3.11283302	1.95584595	-1.77328098
H	2.32071495	1.26183999	-3.18472004
C	2.73313189	-0.26681301	-0.62873399
H	3.10654998	0.27825499	0.24966601

H	3.57120609	-0.32118401	-1.33219695
O	2.34995508	-1.57253397	-0.25256100
C	3.16068292	-2.17534208	0.66173899
O	4.28729916	-1.80404603	0.89442003
N	2.52863407	-3.23090196	1.21963704
H	1.51847696	-3.21577191	1.17848599
C	3.15436912	-3.95052099	2.31087303
H	3.12964392	-3.38177896	3.24585104
H	2.63115597	-4.89623499	2.45303702
H	4.19432402	-4.15623522	2.05868793
H	0.80689901	0.68636900	0.92788100

S8.2. Frequencies

Mode	IR frequency	IR intensity	Raman intensity
1	34.62730000	0.31180000	0.00000000
2	46.75080000	3.68750000	0.00000000
3	69.75420000	2.92330000	0.00000000
4	94.11940000	0.62470000	0.00000000
5	101.82490000	1.35760000	0.00000000
6	109.34970000	19.40240000	0.00000000
7	130.92630000	1.36100000	0.00000000
8	139.40800000	1.18020000	0.00000000
9	143.73380000	1.44730000	0.00000000
10	162.66140000	3.72500000	0.00000000
11	212.95630000	2.67810000	0.00000000
12	218.09650000	5.16600000	0.00000000
13	242.24620000	1.92610000	0.00000000
14	286.49500000	3.70240000	0.00000000
15	290.31600000	9.92170000	0.00000000
16	340.61720000	2.94660000	0.00000000
17	367.09520000	47.09960000	0.00000000
18	396.21240000	15.72550000	0.00000000
19	418.75980000	47.65930000	0.00000000
20	435.93160000	4.99780000	0.00000000
21	471.82860000	8.12700000	0.00000000
22	492.39280000	25.01030000	0.00000000
23	550.40000000	38.18270000	0.00000000
24	563.29190000	55.30780000	0.00000000
25	579.66930000	7.34640000	0.00000000
26	585.61120000	17.31650000	0.00000000
27	619.20690000	6.83260000	0.00000000
28	644.60530000	6.17180000	0.00000000
29	702.04640000	25.60440000	0.00000000
30	733.29000000	9.63030000	0.00000000
31	751.14710000	32.51280000	0.00000000
32	759.93720000	19.52320000	0.00000000
33	798.00590000	5.39070000	0.00000000
34	822.73490000	8.37000000	0.00000000
35	832.53800000	23.99780000	0.00000000
36	932.34520000	17.73150000	0.00000000
37	959.54180000	7.18000000	0.00000000
38	987.31900000	34.60030000	0.00000000
39	993.54360000	4.55130000	0.00000000
40	1000.91080000	14.93530000	0.00000000
41	1005.75000000	0.81560000	0.00000000
42	1025.71950000	38.59420000	0.00000000
43	1063.43310000	14.49030000	0.00000000
44	1070.08960000	15.37270000	0.00000000
45	1096.60090000	4.06150000	0.00000000
46	1138.27960000	14.57620000	0.00000000
47	1153.24640000	15.49640000	0.00000000
48	1183.58710000	10.85470000	0.00000000
49	1195.14590000	92.93310000	0.00000000
50	1204.04770000	15.16680000	0.00000000
51	1221.76470000	23.11590000	0.00000000
52	1255.65870000	10.10970000	0.00000000
53	1289.18710000	198.12380000	0.00000000
54	1300.10270000	2.26720000	0.00000000
55	1326.84230000	299.71940000	0.00000000
56	1343.73110000	29.15070000	0.00000000
57	1398.68020000	30.95810000	0.00000000
58	1414.47140000	2.16090000	0.00000000
59	1418.92540000	7.30440000	0.00000000
60	1442.61920000	32.75010000	0.00000000

61	1465.44970000	11.29290000	0.00000000
62	1475.09690000	0.94880000	0.00000000
63	1484.71020000	28.23630000	0.00000000
64	1485.95330000	10.16550000	0.00000000
65	1495.66200000	20.58370000	0.00000000
66	1505.63060000	11.15460000	0.00000000
67	1512.78420000	10.05000000	0.00000000
68	1591.42260000	248.91230000	0.00000000
69	1615.09900000	41.79760000	0.00000000
70	1655.87060000	28.45490000	0.00000000
71	1675.19270000	647.16300000	0.00000000
72	1707.93970000	4.85100000	0.00000000
73	1831.22930000	384.28860000	0.00000000
74	3019.40160000	12.60520000	0.00000000
75	3040.29400000	25.08450000	0.00000000
76	3045.20070000	51.10800000	0.00000000
77	3068.95120000	12.85120000	0.00000000
78	3106.20900000	10.28800000	0.00000000
79	3125.12850000	22.18460000	0.00000000
80	3142.60920000	11.86580000	0.00000000
81	3162.05000000	8.71740000	0.00000000
82	3195.81840000	6.60720000	0.00000000
83	3210.75250000	7.39350000	0.00000000
84	3224.57250000	10.62090000	0.00000000
85	3240.19260000	5.32310000	0.00000000
86	3614.42810000	102.54770000	0.00000000
87	3729.40110000	18.03030000	0.00000000

S9. CALCULATIONS ON NPPOC (AFTER LOSS OF H^+; S_0)

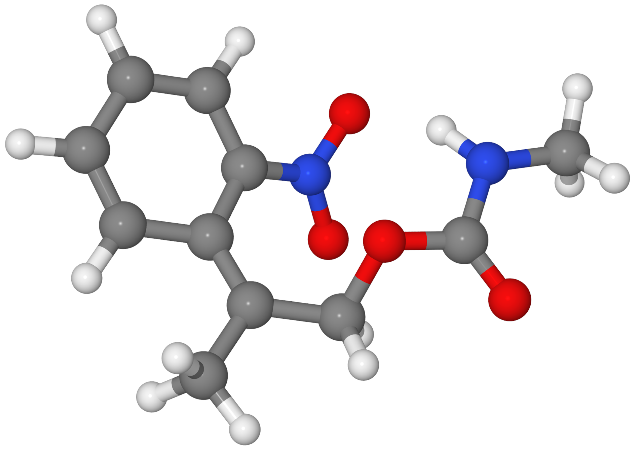

Route	: # opt freq wb97xd/6-311++g(d,p) geom=connectivity int=ultrafine	
SMILES	: C[C](COC(=O)NC)c1ccccc1N(=O)=O	
Formula	: $C_{11}H_{13}N_2O_4^{1-}$	
Charge	: -1	
Multiplicity	: 1	
Energy	: -837.31174759	a.u.
Gibbs Energy	: -837.11966100	a.u.
Number of imaginary frequencies	: 0	

S9.1. Cartesian Co-ordinates (XYZ format)

30

C	-3.73561001	-0.59105700	-0.67162502
C	-3.08367801	0.50276101	-0.20201400
C	-1.65233803	0.50014198	0.09228000
C	-1.10536301	-0.85204899	0.19738400
C	-1.77862000	-1.94854605	-0.39269000
C	-3.06228399	-1.83776402	-0.84478998
H	-4.79218292	-0.51539099	-0.91597497
H	-3.63261700	1.43077803	-0.09381000
H	-1.25726104	-2.89703989	-0.40728399
H	-3.58168602	-2.69565296	-1.25642896
N	0.04816900	-1.14442098	0.90851498
O	0.68758500	-2.20472693	0.64957798
O	0.44773000	-0.37899300	1.80402994
C	-0.96248001	1.68568206	0.11472600
C	-1.69974005	3.00270510	0.01213800
H	-2.49738503	3.09855604	0.75869000
H	-1.01620102	3.84118104	0.17294100
H	-2.16475892	3.15005207	-0.97360301
C	0.52361202	1.80033195	0.17143200
H	0.90908301	1.63708699	1.18703794
H	0.84674799	2.78362298	-0.18094000
O	1.13551795	0.79592001	-0.66407800
C	2.46122789	0.64139199	-0.51564503
O	3.23136592	1.53554904	-0.22336400
N	2.82106709	-0.64592999	-0.78144401
H	2.09518194	-1.32709396	-0.56756699

C	4.18183184	-1.05104995	-0.51062900
H	4.41067791	-1.05109406	0.56187803
H	4.33331394	-2.05785394	-0.90460300
H	4.87511921	-0.36927000	-1.00592196

S9.2. Frequencies

Mode	IR frequency	IR intensity	Raman intensity
1	46.98430000	1.68740000	0.00000000
2	58.46050000	3.09140000	0.00000000
3	66.23500000	4.76870000	0.00000000
4	102.89480000	0.53930000	0.00000000
5	109.28100000	0.86360000	0.00000000
6	121.45630000	8.36330000	0.00000000
7	142.82790000	4.27750000	0.00000000
8	153.88550000	5.13240000	0.00000000
9	163.27960000	2.56260000	0.00000000
10	177.23620000	1.25650000	0.00000000
11	221.70930000	3.28330000	0.00000000
12	241.00320000	1.44650000	0.00000000
13	273.09690000	9.77600000	0.00000000
14	292.55650000	6.40700000	0.00000000
15	303.60960000	1.51350000	0.00000000
16	357.73440000	2.60740000	0.00000000
17	370.61340000	16.33210000	0.00000000
18	417.83970000	19.56920000	0.00000000
19	425.28420000	2.35490000	0.00000000
20	482.04470000	2.45490000	0.00000000
21	502.86330000	32.89770000	0.00000000
22	576.61440000	2.36280000	0.00000000
23	578.52260000	1.78960000	0.00000000
24	590.06780000	11.09430000	0.00000000
25	649.47120000	78.95900000	0.00000000
26	676.97180000	34.75870000	0.00000000
27	711.53960000	31.83850000	0.00000000
28	728.05120000	53.42250000	0.00000000
29	753.69900000	12.31930000	0.00000000
30	775.21290000	18.97200000	0.00000000
31	784.03900000	5.37910000	0.00000000
32	803.44140000	12.24150000	0.00000000
33	832.50660000	22.66940000	0.00000000
34	864.69740000	5.18930000	0.00000000
35	946.99630000	15.99940000	0.00000000
36	974.26520000	10.79610000	0.00000000
37	975.22190000	2.30050000	0.00000000
38	978.60560000	2.00250000	0.00000000
39	991.46240000	10.56810000	0.00000000
40	1024.78310000	77.23900000	0.00000000
41	1037.78210000	20.73050000	0.00000000
42	1048.77220000	12.97970000	0.00000000
43	1084.36790000	6.67450000	0.00000000
44	1116.38320000	18.91130000	0.00000000
45	1150.22850000	13.08070000	0.00000000
46	1170.07820000	14.05690000	0.00000000
47	1190.58560000	59.39310000	0.00000000
48	1198.00300000	84.31730000	0.00000000
49	1202.55020000	5.69740000	0.00000000
50	1240.72360000	54.04840000	0.00000000
51	1265.31670000	305.78830000	0.00000000
52	1275.78850000	59.00980000	0.00000000
53	1319.92640000	38.68630000	0.00000000
54	1327.55670000	1119.55060000	0.00000000
55	1401.16270000	224.90480000	0.00000000
56	1408.41660000	3.62900000	0.00000000
57	1428.36170000	35.35870000	0.00000000
58	1438.79450000	200.90880000	0.00000000
59	1455.14180000	82.42190000	0.00000000
60	1462.95150000	62.46390000	0.00000000

61	1485.44230000	33.65850000	0.00000000
62	1490.64670000	9.40080000	0.00000000
63	1493.19800000	2.54460000	0.00000000
64	1500.13180000	19.02300000	0.00000000
65	1511.85160000	4.89180000	0.00000000
66	1514.81950000	19.33620000	0.00000000
67	1569.50930000	53.64440000	0.00000000
68	1601.39030000	244.70060000	0.00000000
69	1610.23700000	86.92050000	0.00000000
70	1674.49300000	95.78260000	0.00000000
71	1792.01630000	456.07470000	0.00000000
72	2986.41730000	151.23080000	0.00000000
73	3013.41020000	32.22700000	0.00000000
74	3024.31470000	82.54030000	0.00000000
75	3041.60700000	54.28990000	0.00000000
76	3081.52290000	20.79150000	0.00000000
77	3097.53060000	53.97730000	0.00000000
78	3099.90520000	42.90470000	0.00000000
79	3138.07380000	22.01210000	0.00000000
80	3154.42940000	32.45730000	0.00000000
81	3189.97700000	29.77970000	0.00000000
82	3205.18250000	30.41750000	0.00000000
83	3227.01860000	12.54300000	0.00000000
84	3501.56360000	269.89310000	0.00000000

S10. CALCULATIONS ON NPPOC (DISSOCIATED; S_0)

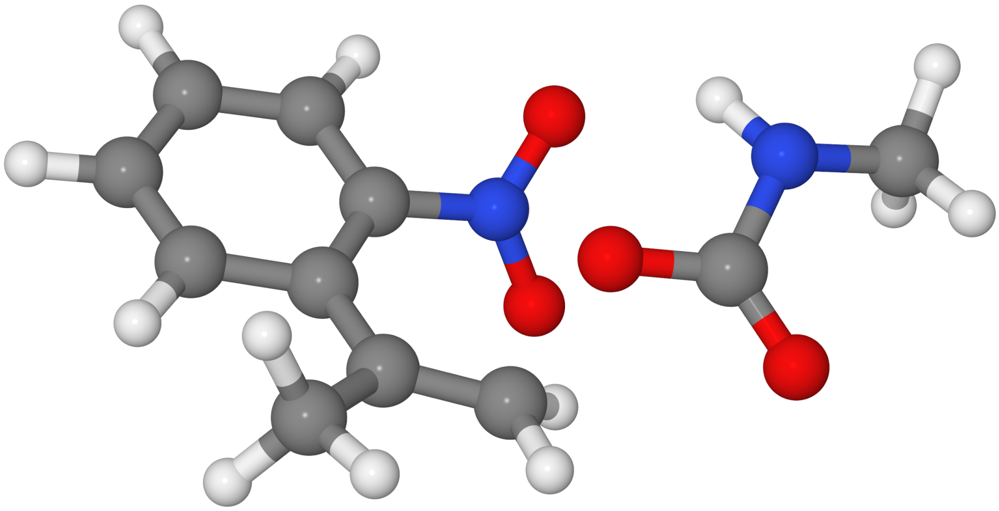

Route : # opt=modredundant wb97xd/6-311++g(d,p) geom=connectivity int=ultrafine
SMILES : CC(COC(=O)NC)c1ccccc1N(=O)=O
Formula : $C_{11}H_{13}N_2O_4$
Charge : -1
Multiplicity : 1
Energy : -837.291387427 a.u.

S10.1. Cartesian Co-ordinates (XYZ format)

30

C	-3.97369500	-0.24938000	-0.54895000
C	-3.10464200	0.76240400	-0.21512700
C	-1.73967300	0.56335400	0.17312100
C	-1.39324100	-0.82335300	0.22587200
C	-2.25842700	-1.84543300	-0.16506000
C	-3.55730500	-1.58415400	-0.54684600
H	-4.99435400	0.00175500	-0.82298400
H	-3.47513700	1.77776000	-0.24901500
H	-1.88242900	-2.86078000	-0.13837500
H	-4.22791000	-2.38969600	-0.82106900
N	-0.11897500	-1.33306900	0.70901500
O	0.44228800	-2.20251300	0.05081700
O	0.29526600	-0.94013100	1.78800600
C	-0.90137200	1.71902800	0.30432600
C	-1.42428000	3.02506300	-0.26267600
H	-2.25199200	3.45252500	0.32127800
H	-0.62395800	3.76828200	-0.26620100
H	-1.78079900	2.92538400	-1.29763800
C	0.43207200	1.69671400	0.69919300
H	0.78920400	0.99243500	1.43480500
H	0.96824700	2.63806300	0.70097600
O	1.44783100	0.84596900	-0.66259500
C	2.68210100	0.60363900	-0.37057300

O	3.42312600	1.29060200	0.33437400
N	3.15511700	-0.57401300	-0.96170900
H	2.40259900	-1.21843400	-1.15562700
C	4.37690600	-1.16099200	-0.46145900
H	4.27570800	-1.56665500	0.55526000
H	4.69354200	-1.96631400	-1.13130800
H	5.15552800	-0.39750400	-0.43698500
